# Taurine Reprograms Mammary-Gland Metabolism and Alleviates Inflammation Induced by *Streptococcus uberis* in Mice

**DOI:** 10.3389/fimmu.2021.696101

**Published:** 2021-06-10

**Authors:** Riguo Lan, Zhixin Wan, Yuanyuan Xu, Zhenglei Wang, Shaodong Fu, Yuanyuan Zhou, Xinguang Lin, Xiangan Han, Zhenhua Luo, Jinfeng Miao, Yulong Yin

**Affiliations:** ^1^ Ministry of Education Joint International Research Laboratory of Animal Health and Food Safety, Key Laboratory of Animal Physiology & Biochemistry, College of Veterinary Medicine, Nanjing Agricultural University, Nanjing, China; ^2^ Shanghai Veterinary Research Institute, Chinese Academy of Agricultural Sciences, Shanghai, China; ^3^ School of Water, Energy & Environment, Cranfield University, Cranfield, United Kingdom; ^4^ Chinese Academy of Science, Institute of Subtropical Agriculture, Research Center for Healthy Breeding Livestock & Poultry, Hunan Engineering & Research Center for Animal & Poultry Science, Key Laboratory of Agroecology in Subtropical Region, Scientific Observing and Experimental Station of Animal Nutrition and Feed Science in South-Central China, Ministry of Agriculture, Changsha, China

**Keywords:** mastitis, *Streptococcus uberis*, taurine, metabolic regulation, AMPK, mTOR

## Abstract

*Streptococcus uberis* (*S. uberis*) is an important pathogen causing mastitis, which causes continuous inflammation and dysfunction of mammary glands and leads to enormous economic losses. Most research on infection continues to be microbial metabolism-centric, and many overlook the fact that pathogens require energy from host. Mouse is a common animal model for studying bovine mastitis. In this perspective, we uncover metabolic reprogramming during host immune responses is associated with infection-driven inflammation, particularly when caused by intracellular bacteria. Taurine, a metabolic regulator, has been shown to effectively ameliorate metabolic diseases. We evaluated the role of taurine in the metabolic regulation of *S. uberis*-induced mastitis. Metabolic profiling indicates that *S. uberis* exposure triggers inflammation and metabolic dysfunction of mammary glands and mammary epithelial cells (the main functional cells in mammary glands). Challenge with *S. uberis* upregulates glycolysis and oxidative phosphorylation in MECs. Pretreatment with taurine restores metabolic homeostasis, reverses metabolic dysfunction by decrease of lipid, amino acid and especially energy disturbance in the infectious context, and alleviates excessive inflammatory responses. These outcomes depend on taurine-mediated activation of the AMPK–mTOR pathway, which inhibits the over activation of inflammatory responses and alleviates cellular damage. Thus, metabolic homeostasis is essential for reducing inflammation. Metabolic modulation can be used as a prophylactic strategy against mastitis.

## Introduction

Mastitis is a highly prevalent and important infectious disease worldwide. More than 150 different pathogens can cause mammary gland infection and no specific vaccines are available (1). Antibiotics combined with anti-inflammatory drug therapy is the usual method to control mastitis (2). These therapies are often ineffective for the diversity of pathogens and the complexity of mammary tissue (3, 4). Milk production of therapeutically treated cows will often decline significantly during subsequent lactations necessitating culling of treated animals. Moreover, the extensive use of antibiotics in the treatment and control of mastitis has implications for human health *via* residual drugs and increased emergence of antibiotics-resistant bacteria strains then enter the food chain (5). Mastitis leads to great economic losses to the dairy industry and poses serious potential threats to public health. Clearly, new and innovative approaches for mastitis control are needed.

*S. uberis* accounts for approximately 33 percent of the isolates obtained from clinical cases of bovine mastitis (6). Growing evidence suggests that it can also infect humans thus directly threatening human health (7). Mastitis induced by *S. uberis* is difficult to control because it may internalize and persist in mammary cells to develop an intracellular infection, thereby escaping detection and subsequent elimination by the host immune system and/or antibiotics (8). In the past decade, our lab focused on the interaction of pathogen infection and mammary defense, with the purpose of developing an alternative for prevention mastitis (9–11). We found that immunophysiological regulators (amino acids, polysaccharide, vitamins, and antioxidants, etc.) can reduce inflammatory damage of mammary tissues by improving the disease resistance of the host animal (9–13). Physiological regulation of the immune system has potential as a putative therapeutic and prophylactic strategy for mastitis control with promising clinical applications.

Taurine is one of the most abundant free amino acids in most animal tissues and plays an important role in several essential biological processes. Our previous studies have established that taurine administration can modulate bacterial mammary infections including those due to *S. uberis* (12–14). We wonder how cells sense nutrient signaling from taurine and change it to an anti-infection effect *in vitro* and *in vivo*. Taurine downregulates *S. uberis* induced inflammation and decreases intracellular bacteria through the PI3K (phosphoinositide 3-kinase)/Akt (also known as PKB, protein kinase B)/mTOR (mammalian target of rapamycin), PIs (phosphoinositides)/PLCγ (phospholipase C γ)/DG (diacylglycerol)/PKCα (protein kinase C α), and IP3 (inositol triphosphate)/Ca^2+^/CaM (calmodulin)/CaN (calcineurin) pathways by activating TLRs (toll-like receptors) (14–16). Taurine also upregulates autophagy of MECs (mammary epithelial cells) during *S. uberis* infection (1). We postulate that taurine attenuates *S. uberis-*induced inflammation through its regulatory effect on metabolism.

Host metabolic homeostasis is critical to disease progression during pathogen infection, as intracellular bacteria often reprogram host metabolism to divert nutrients for their own growth. Although pathogenic microorganisms also change their metabolic pathways to facilitate invasion, the metabolic disorders of host are often closely related to diseases. For example, *Mycobacterium tuberculosis* increases glucose uptake and decreases oxidative phosphorylation (OXPHOS) to redirect glycolytic intermediates toward lipid synthesis, which facilitates bacterial growth within macrophages (17, 18). Metabolic intervention can be used to combat pathogenic infections. Wang et al. show that limiting glucose utilization significantly improves tolerance to cerebral malaria (19). *In vivo*, taurine participates in the synthesis of fatty acids and steroids (20). It can be converted into taurocholic acid and participate in bile formation thus promoting the emulsification and absorption of lipids (20). Taurine may therefore influence *S. uberis* infection *via* regulation of cell metabolism.

Metabolic homeostasis is primarily regulated by the precise coordination of glycolysis, the TCA cycle, the pentose phosphate pathway, fatty acid oxidation, fatty acid synthesis, and amino acid metabolism. Recently, it had been discovered that various immune cells undergo distinct metabolic changes in the face of pathogenic infections. These mechanisms require an increased energy expenditure and the consumption of intermediate metabolites (21–23). M1 macrophages, T helper 1 (TH1) and T helper 17 (TH17) cells, upregulate glycolysis which helps define their inflammatory phenotype (24). M2 macrophages as well as quiescent and regulatory T cells, may be characterized as an anti-inflammatory phenotype depending on OXPHOS and fatty acid oxidation for tissue repair and anti-inflammatory cytokine production (25). MECs are responsible for milk synthesis. It has been established that they are involved in innate immune and anti-infection responses (26, 27). The metabolic changes and underlying mechanisms of MECs as a result of bacterial infection remain unclear.

AMP-dependent protein kinase (AMPK) is an “energy receptor” that regulates energy metabolism in eukaryotic cells (28). mTOR is a “hub” that regulates metabolism and is targeted by AMPK activation (29). These molecules communicate in the cellular metabolism and energy production. Whether taurine regulates the metabolism of the host through them and affects the redistribution of cellular metabolite and energy in the mammary glands, thereby regulating the infection of *S. uberis*, is of interest. Mouse is a common animal model for studying bovine mastitis (30, 31). Herein, we provide insight into the metabolic changes of mouse mammary glands/cells in *S. uberis* infection and the regulatory mechanism(s) of taurine in this bioprocess. Our results contribute toward developing novel prophylactic strategies for mastitis and possibly other intracellular infections.

## Materials and Methods

### Bacterial Strains and Growth Conditions

*S. uberis* (0140J strain) was inoculated into Todd–Hewitt broth medium containing 2% fetal bovine serum (FBS, Gibco, USA) and cultured at 37°C in an orbital shaker to log-phase growth (OD_600_ = 0.4–0.6). *E. coli* (NJ-17 strain) was inoculated into Luria–Bertani medium at 37°C and cultured in an orbital shaker to log-phase growth (OD_600_ = 0.5–0.6).

### Mouse Infections

Female C57BL/6J mice (6–8 weeks old) were used for the animal experiments. All procedures involving animals were approved by the committee on the Use and Care of Animals of Nanjing Agricultural University (Nanjing, China). Our protocol number approved by the animal welfare committee is PZ2019144. Twenty-four healthy pregnant mice were housed in individual cages and provided water and food *ad libitum*. Following acclimatization, the mice were randomly divided into 4 groups (Control, Taurine, *S. uberis*, and Taurine + *S. uberis* groups); each group contained 6 mice. Starting on gestation day 14, 100 mg/kg taurine (dissolved in sterile pyrogen-free saline), or an equal volume of saline, was administered daily to each mouse by gavage until parturition. At 72 h after parturition, mice in the *S. uberis* and Taurine + *S. uberis* groups were infused with 100 CFUs *S. uberis* in 50 μL into the L4 and R4 teats. The offspring were weaned 2 h prior to this infusion. Following administration of ether anesthesia, the L4 and R4 teats were moistened with 75% ethanol, and a 33-gauge needle (fitted to a 1-mL syringe) was gently inserted into the mammary duct, after which 50 μL *S. uberis* was slowly infused. At 24 h PI, all mice in the 4 groups were euthanized, and the mammary glands were aseptically collected and stored at −80°C until analysis.

### EpH4-Ev Cell Culture and Treatment

EpH4-Ev cells (mouse mammary epithelial cell line) were grown at 37°C in DMEM supplemented with 10% FBS in 6-well plates until they reached 70–80% confluency. The resulting monolayers were cultured in FBS-free DMEM for 4 h and then further cultured in FBS-free DMEM for 24 h, with or without 45 mmol/L taurine. Next, both groups of cells were infected with *S. uberis* at an MOI of 10 as before(16) for 1, 2, or 3 h for metabolomics analysis. In other experiments, EpH4-Ev cells were exposed to *S. uberis* for 3 h.

Cell samples prepared for GC–TOF-MS analysis were collected as described previously (32). Briefly, cells (plated at approximately 5 × 10^6^ cells/well) were infected with *S. uberis* for 1, 2, or 3 h. The cell supernatants were discarded, and the cells were washed three times with cold phosphate-buffered saline (PBS), quenched with 400 µL cold methanol (precooled at -80°C), and held at -80°C for 30 min. An additional 400 µL double distilled water was added to the plates, and the cells were scraped off separate plates. The cell suspensions were stored at -80°C and then used to prepare samples for metabolomics analysis.

For extracellular flux analysis, EpH4-Ev cells were challenged with 10 μg/mL LPS for 12 h, or with inactivated *S. uberis* or *E. coli* NJ-17 at a MOI of 100 for 3 h, at 37°C.

For the inhibition and activation experiments, cells were pretreated as described below before *S. uberis* infection. Specifically, EpH4-Ev cells were grown at 37°C in DMEM with 10% FBS in 6-well plates and grown to 70–80% confluency. After culturing the cells in FBS-free DMEM for 4 h, the monolayers were treated with 5 mM 2-DG (Selleckchem, USA) for 1 h, 25 μM CPI-613 (Selleckchem, USA) for 12 h, 100 nM MHY1485 (Selleckchem, USA) for 24 h, or 10 µM Compound C (Selleckchem, USA) for 1 h. All the inhibitors or activators used in this study had been determined by cell viability and they were nonpoisonous to cells.

### Culturing and Treating RAW 264.7 Macrophages

For extracellular flux analysis, RAW 264.7 macrophages were grown to 70–80% confluency in DMEM containing 10% FBS in XF 24-well plates. Next, the cells were stimulated with 100 ng/mL LPS for 12 h or with inactivated *S. uberis* or *E. coli* NJ-17 at an MOI of 5 for 3 h, at 37°C.

### Metabolite Extraction for GC–TOF-MS

A 50 ± 1 mg sample of each mammary gland was extracted with 450 μL extraction buffer (V_Methanol_: V_Chloroform_ = 3: 1) and placed into 2-mL EP tubes. Next, 10 µL l-2-chlorophenylalanine (1 mg/mL stock in dH_2_O) was added to each mammary gland sample and vortexed for 30 s. Subsequently, 10 µL adonitol (0.5 mg/mL stock in dH_2_O) was added to the cell samples, followed by vortexing again for 30 s. The vortexed samples were homogenized for 4 min then ultrasonicated for 5 min while maintained in an ice-water bath. The ultrasonicated samples were centrifuged for 15 min at 5,000 × *g* at 4°C. Next, approximately 350 μL supernatant from the mammary-gland samples, or 600 μL supernatant from the cell samples, was transferred into fresh 1.5 mL EP tubes; 40 μL supernatant from each sample was pooled for quality control. The samples were dried completely in a vacuum concentrator without heating, and each mammary gland sample or cell sample was resuspended in 60 or 20 μL methoxyamine hydrochloride (20 mg/mL in pyridine), respectively. After incubation for 30 min at 80°C, approximately 80 µL *N*, *O*-Bis (trimethylsilyl) trifluoroacetamide (BSTFA) reagent (1% trimethylchlorosilane (TMCS), v/v) was added to the sample aliquots and incubated for 1.5 h at 70°C before GC–TOF-MS analysis.

### GC–TOF-MS Analysis

GC–TOF-MS analyses were performed using an Agilent 7890 GC instrument coupled with a Pegasus 4D TOF-MS instrument. This system utilized a DB-5MS capillary column coated with 5% diphenyl cross-linked with 95% dimethylpolysiloxane (30 m × 250 μm [inner diameter], 0.25-μm film thickness; J&W Scientific, Folsom, CA, USA). Next, 1-μL aliquots of the analytes were injected in splitless mode. Helium was used as the carrier gas, the front inlet purge flow was 3 mL/min, and the gas flow rate through the column was 1 mL/min. The initial temperature was maintained at 80°C for 1 min, raised to 290°C at a rate of 10°C/min, and then maintained for 8 min at 290°C. The injection, transfer line, and ion-source temperatures were 280, 295, and 220°C, respectively. Ionization was performed at -70 eV in electron-impact mode. MS data were acquired in full-scan mode with a mass/charge range of 50 to 600, at a rate of 10 spectra/s after a solvent delay of 7.9 min.

### RNA Extraction and qRT-PCR

Total RNA was extracted using TRIZOL reagent (Invitrogen, Carlsbad, CA, USA) and reverse transcribed (RT) into cDNA using PrimeScriptTM RT reagent kit (Takara, Dalian, China). The PCR reaction was in a total volume of 20 µL using a SYBR Premix Ex TaqTM (Takara, Dalian, China) in which 2 µL cDNA was added as a template. The primer sequences are in **Supplementary Table 1**. As an internal control, the same RT products were subjected to PCR in the presence of a second pair of primers specific to β-actin. Analysis of the relative dates of gene expression used the 2^-△△Ct^ method.

### Detection of Relative Enzyme Activities

The hexokinase (HK), phosphofructokinase (PFK), lactate dehydrogenase (LDH), pyruvate dehydrogenase (PDH), succinate dehydrogenase (SDH), mitochondrial complex IV, glutamate dehydrogenase (GDH), glutamic oxalacetic transaminase (GOT), and glutamic-pruvic transaminase (GPT) (Solarbio, Beijing, China), and N-acetyl-β-D-glucosaminidase (NAGase) (Jiancheng, Bioengineering Institute, Nanjing, China) activities in EpH4-Ev cells was measured using commercial kits according to the instructions of respective manufacturers.

### Extracellular Flux Analysis

Real-time extracellular acidification rate (ECAR) and oxygen consumption rate (OCR) values of EpH4-Ev cells or RAW 264.7 cells were analyzed using an XF-24 Extracellular Flux Analyzer (Seahorse Bioscience, MA, USA). Briefly, the basal metabolic rates of cells seeded in quintuplicate were determined using 4 consecutive measurements in unbuffered, sterile-filtered Seahorse medium (8.3 g DMEM powder, 0.016 g phenol red, and 1.85 g NaCl in 1 L Milli-Q water, pH = 7.4 at 37°C) containing 2 mM glutamine (for ECAR measurements) or 10 mM glucose, 2 mM glutamine, and 1 mM sodium pyruvate (for OCR measurements). The cells were incubated in a CO_2_-free incubator at 37°C for 1 h before measurements. After 3 basal measurements, 3 consecutive measurements were acquired after the different treatments. For ECAR detection, the cells were sequentially exposed to 10 mM glucose, 2 mM oligomycin, and 50 mM 2-DG. For OCR determinations, the cells were sequentially exposed to 2 mM oligomycin, 1 mM FCCP, and 2 µM antimycin A + 1 µM rotenone. Next, 20 mM glucose and 1 mM pyruvate were added along with FCCP to fuel maximal respiration. All compounds used during the Seahorse runs were acquired from Sigma-Aldrich. The cell number in each well was normalized by measuring the total protein content, using a Synergy™ H1 Hybrid Multi-Mode Microplate Reader (BioTek Instruments). The 24-well microplates used for Seahorse measurements were pretreated with Cell-Tak Cell and Tissue Adhesive (Corning).

### ELISA Testing

TNF-α and IL-1β levels in EpH4-Ev cells were measured using ELISA kits (Rigor Bioscience, Beijing, China), according to the manufacturer’s instructions. The detection limit of IL-1β ELISA kit is 0-160 ng/L and the detection limit of IL-1β ELISA kit is 0-40 ng/L.

### ROS Measurements

Per the manufacturer’s instructions of the ROS assay kit (Beyotime, Nantong, China), EpH4-Ev cells were incubated in 10 μmol/L DCFH-DA for 30 min at 37°C, washed 3 times in PBS, and detached with trypsin. The cells were centrifuged at 400 × *g* for 5 min, resuspended in PBS, and immediately analyzed by flow cytometry using a FACSCanto instrument; 10,000 cells/sample were analyzed using CellQuest Pro acquisition software and FlowJo software.

### Western Blotting

Intracellular protein levels were determined by western blotting. An anti-β-actin antibody (Bioworld, USA) was used as a loading control. Cells were washed twice in ice-cold PBS and lysed by incubation in RIPA buffer (Beyotime, Nantong, China) containing PMSF (Beyotime, Nantong, China) on ice for 30 min. Supernatants were collected by centrifuging at 5000 × *g* for 10 min at 4°C, and protein concentrations were determined using a Bicinchoninic Acid Assay Kit (Beyotime, Nantong, China) and detected with a spectrophotometer (Tecan, Männedorf, Switzerland). Proteins were separated by electrophoresis on a polyacrylamide gel and transferred to polyvinylidene fluoride membranes (Millipore, USA). The membranes were blocked with 5% non-fat milk diluted in Tris buffered saline with Tween-20 (TBST) for 2 h at room temperature (approximately 10–25°C) and hybridized overnight with an appropriate primary antibody at 4°C. Primary antibodies were diluted in TBST as follows: β-actin (Bioworld, BS6007M), AMPKα (CST, USA, 2532), p-AMPK (CST, USA, 2535), mTOR (CST, USA, 2972S), p-mTOR (CST, USA, 5536), p70 S6K (CST, USA, 2708), p-p70 S6K(CST, USA, 9205), 4E-BP1 (CST, USA, 9644), and p-4E-BP1 (CST, USA, 2855). The membranes were washed three times with TBST before and after incubation with a horseradish peroxidase (HRP)-linked anti-rabbit IgG (CST, USA, 7074S) secondary antibody at room temperature (approximately 10-25°C) for 2 h. Signals were detected using an ECL Western Blot Analysis System (Tanon, Shanghai, China). Bands were quantified using ImageJ software (NIH, USA).

### Statistical Analysis

Statistical analysis was performed using GraphPad Prism 7 software. All data are represented as the mean ± SEM. To meet the assumption of homogeneity of variance, an analysis of variance (ANOVA) was performed, followed by Tukey’s multiple comparison test. The Student’s t-test was used to compare the difference between two groups, and differences were considered statistically significant at p < 0.05.

## Results

### Taurine Reprograms *S. uberis*-Induced Metabolic Changes in Mammary Glands

**Figure 1:**
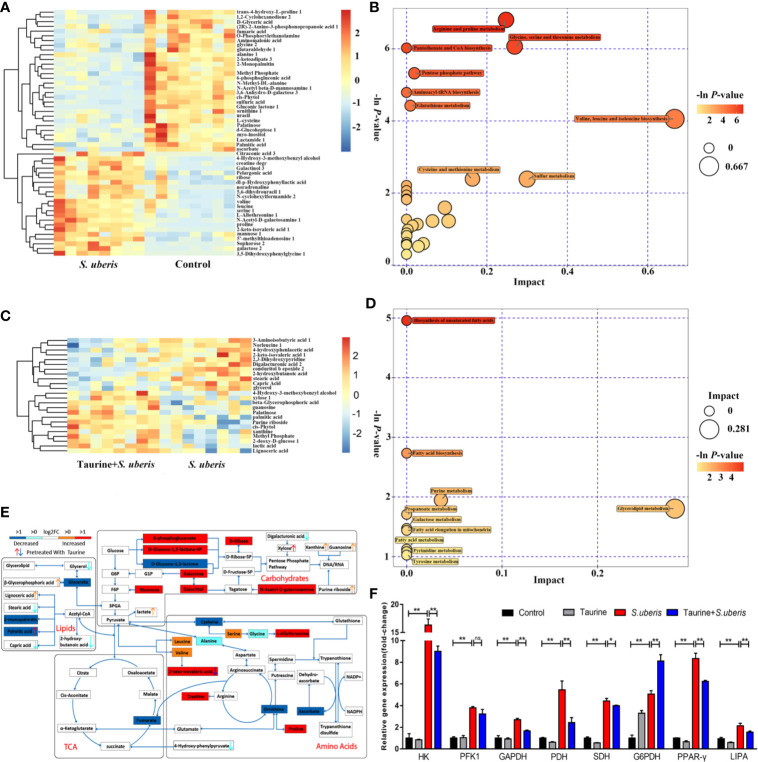
Taurine relieves *S. uberis*-induced metabolic changes in mammary glands. Pregnant C57BL/6J mice were administered 100 mg/kg taurine (in sterile pyrogen-free saline) or an equal volume of saline by daily gavage until parturition. At 72 h post-parturition, mice in the *S. uberis* and Taurine + *S. uberis* groups were infusedwith 100 colony-forming units (CFUs) of *S. uberis* in 50 μL sterile saline into the left 4th (L4) and right 4th (R4) teats. At 24 h post-*S. uberis*-infusion (PI), mammary glands were collected for gas chromatography–time-of-flight-mass spectrometry (GC-TOF-MS) analysis. Eight mice of each group were sampled (n = 8). **(A)** Significant changes in metabolites in C57BL/6J mice mammary glands (*S. uberis* group versus control group) are shown in the heatmap. **(B)** Metabolome map of significant metabolic pathways of mammary gland metabolites (*S. uberis* group versus control group). The x-axis represents pathway enrichment, and the y-axis represents the pathway impact. Large sizes and dark colors represent major pathway-enrichment and high pathway-impact values, respectively. **(C)** Significant metabolic changes in C57BL/6J mice mammary glands are represented in the heatmap (Taurine + *S. uberis* group versus *S. uberis* group). **(D)** Metabolome map of significant metabolic pathways as indicated by metabolites in C57BL/6J mice mammary tissues (Taurine + *S. uberis* group versus *S. uberis* group). **(E)** Model of how taurine changes metabolomic responses to *S. uberis* infection in C57BL/6J mice mammary glands. **(F)** Relative gene expression of mammary gland samples from different groups. Data are represented as mean ± SEM (n = 3). *P < 0.05, **P < 0.01, ns, no significance.

To determine whether taurine regulates metabolism in *S. uberis* induced mastitis, we infected the teats of taurine-pretreated mice with *S. uberis* for 24 h and then collected mouse mammary glands for metabolomic analysis. As shown in **Supplementary Figure 1**, intragastric administration of taurine alone could partly change mammary gland metabolism in mice. The metabolites increased by taurine administration focused on amino acids (such as proline, isoleucine, tyrosine, L-cysteine, L-allothreonine), nucleic acids (xanthine, uridine monophosphate), and taurine-associated metabolite sulfuric acid. These metabolites mainly concentrated on aminoacyl-tRNA biosynthesis, sulfur metabolism, purine metabolism, valine, leucine and isoleucine biosynthesis, and cysteine and methionine metabolism (**Supplementary Figure 1**). Further, taurine administration significantly changed the metabolites in *S. uberis*-infected mammary glands (**Supplementary Figure 2**). *S. uberis*-infected mammary glands exhibited fifty-two different metabolites, including seven upregulated carbohydrate-related metabolites (galactose, mannose, galactinol, N-acetyl-D-galactosamine, 6-phosphogluconate, glucoheptonate, ribose, and 5’-methylthioadenosine) and 7 amino acids or amino-acid derivatives (valine, proline, serine, leucine, L-allothreonine, creatine, and 3, 5-dihydroxyphenylglycine). One TCA cycle intermediate (fumarate), three amino-acid metabolites (cysteine, ornithine and ascorbate) and three fatty acid-metabolism precursors (glycerate, 2-monpalmitin and palmitic acid) are down-regulated (**Figure 1A**). These metabolites were primarily involved in the pentose phosphate pathway; pantothenate and CoA biosynthesis; arginine and proline metabolism; glycine, serine, and threonine metabolism; valine, leucine, and isoleucine biosynthesis; glutathione metabolism and aminoacyl-tRNA biosynthesis (**Figure 1B**). Taurine pretreatment decreased glycerol, stearic acid, capric acid and 2-hydroxybutanoate levels, elevated several lipid metabolites (palmitic acid, lignoceric acid, and β-glycerophosphoric acid) (**Figure 1C**), and influenced unsaturated fatty-acid and fatty-acid biosynthesis, fatty-acid elongation, and purine and pyrimidine metabolism (**Figure 1D**). Integrated metabolite-map and pathway analysis showed lower metabolite levels of the TCA cycle (fumarate) and glutathione metabolism (cysteine, ornithine and ascorbate), along with higher metabolite levels in the pentose phosphate pathway and higher amino acid levels following *S. uberis* infection. These data denoted consumption of more energy and production of anti-infectious metabolites (such as glutathione, an antioxidant) in mammary tissue to resist *S. uberis* challenge (**Figure 1E**). Taurine pretreatment mainly reduced fatty acid metabolism (2-hydroxybutanoate), promoted unsaturated fatty acid and fatty acid biosynthesis, and nucleic acid metabolism (**Figure 1E**). To confirm whether these metabolic changes were reflected at a transcriptional level, we measured the expression of key genes involved in glycolysis (HK, PFK1 and GAPDH), TCA cycle (PDH, SDH), fatty acid metabolism (PPAR-γ, LIPA) and pentose phosphate pathway (G6PDH). In line with our findings, expression of these genes in mammary glands was elevated by *S. uberis* challenge. Taurine dropped off most of the gene expression in *S. uberis* infection but increased gene expression of G6PDH (**Figure 1F**). These data suggest that taurine reprograms metabolism in *S. uberis*-infected mammary glands.

### Taurine Attenuates Metabolic Disturbances in *S. uberis* Challenged MECs

MECs are the main functional cells in mammary glands and play important roles in the mammary gland defense system. Pretreatment with taurine alone changed taurine-associated metabolites in mouse mammary epithelial cell line (EpH4-Ev cells). β-alanine, a competitive inhibitor of taurine transporter (TauT), was decreased by taurine pretreatment. Differently, pretreatment with taurine increased intracellular taurine and taurine-associated metabolites (glutathione, sulfuric acid, etc.). These metabolites belonged to several taurine metabolism related pathways, including sulfur metabolism, taurine and hypotaurine metabolism, β-alanine metabolism, glutathione metabolism, and primary bile acid biosynthesis (**Supplementary Figure 3**).

To investigate whether taurine regulates MECs metabolism during *S. uberis* infection, EpH4-Ev cells were incubated with *S. uberis* for varying times and the intracellular metabolite profile was examined. Metabolites in infected and control cells formed separate clusters on PCA and OPLA-DA plots (**Supplementary Figures 4A–F**) and had significant changes on volcano plots over time (**Supplementary Figures 4G, H**), especially at 3 h post-infection (**Supplementary Figure 4I**). Thus, challenge with *S. uberis* altered the cellular metabolic profile. Taurine pretreatment altered the metabolic profile of *S. uberis*-infected cells over these time points (**Supplementary Figures 4J–R**).

**Figure 2:**
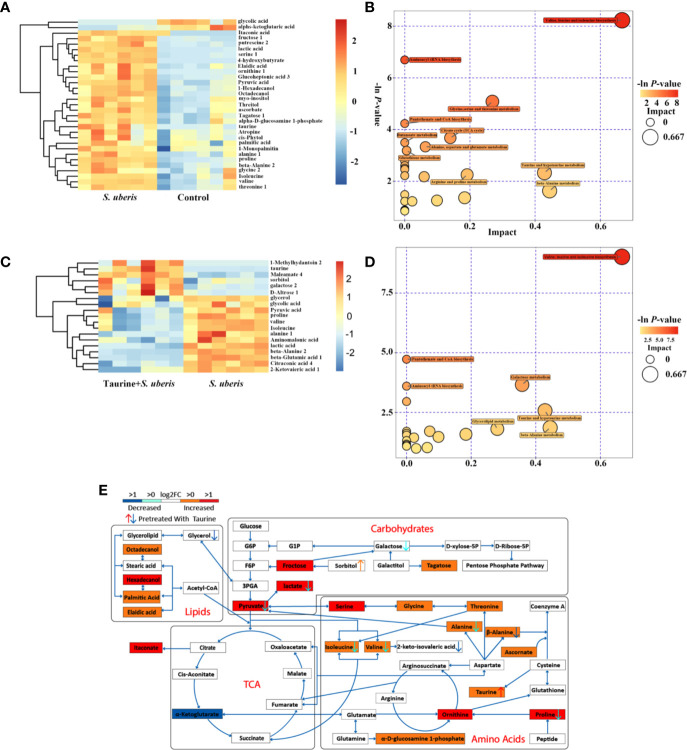
Taurine attenuates metabolic disturbances in *S. uberis* infected MECs. EpH4-Ev cells were pretreated with taurine for 24 h and infected with *S. uberis* in mid-exponential phase at a multiplicity of infection (MOI) of 10 for 3 h at 37°C. Cellular metabolites were extracted and assayed by GC-TOF-MS. Six samples from each group were tested (n = 6). **(A)** Significant changes in metabolites in EpH4-Ev cells (*S. uberis* group versus control group) are shown in the heatmap. **(B)** Metabolome map of significant metabolic pathways reflected by metabolites of EpH4-Ev cells (*S. uberis* group versus control group). The x-axis represents pathway enrichment, and the y-axis represents the pathway impact. Large sizes and dark colors represent major-pathway enrichment and high pathway-impact values, respectively. **(C)** Significant metabolic changes in EpH4-Ev cells (Taurine + *S. uberis* group versus *S. uberis* group) are shown. **(D)** Metabolome map of significant metabolic pathways identified by metabolites in EpH4-Ev cells (Taurine + *S. uberis* group versus *S. uberis* group). **(E)** Model of how taurine alters the metabolomic response of *in vitro* EpH4-Ev cells in response to a 3 h *S. uberis* infection.

Metabolic changes induced by *S. uberis* infection at 1 h was slight and only several metabolites changed, but an increase of glucose-6-phosphate was present (**Supplementary Figures 5A–E**). In contrast, 2 or 3 h of infection with *S. uberis* upregulated three glycolysis-related metabolites (glucose, pyruvate and lactate), four lipid-related metabolites (octadecanol, 1-hexadecanol, 1-monopalmitin, and palmitic acid), ten amino acids (alanine, valine, glycine, threonine, isoleucine, proline, serine, beta-alanine, taurine, and ornithine), and several other carbohydrate-related metabolites (fructose, tagatose, and inositol), while the levels of citrate and α-ketoglutarate were downregulated (**Figure 2A** and **Supplementary Figure 6A**). The alterations at 2 or 3 h post-infection included the following metabolic pathways: aminoacyl-tRNA biosynthesis; TCA cycle; pyruvate metabolism; glycolysis or gluconeogenesis; valine; leucine, and isoleucine biosynthesis; pantothenate and CoA biosynthesis; glycine, serine, and threonine metabolism; arginine and proline metabolism; alanine, aspartate, and glutamate metabolism; and glutathione metabolism (**Figure 2B** and **Supplementary Figure 6B**).

Pretreatment with taurine at 1 h post-infection exerted a slight change in amino acid metabolism (**Supplementary Figures 5C–E**). In *S. uberis*-infected cells (2 or 3 h), taurine pretreatment decreased carbohydrate-related metabolite levels (glucose, pyruvate, lactate, citrate, α-ketoglutarate etc.) and amino acid levels (aspartate, alanine, isoleucine, β-alanine, valine, glutamate, proline), and attenuated their matched pathways (i.e., valine, leucine, and isoleucine biosynthesis; β-alanine, alanine, aspartate, and glutamate metabolism; and TCA cycle (**Figures 2C–E** and **Supplementary Figures 6C–E**). These data suggest that taurine reprograms *S. uberis*-induced metabolic changes in MECs infected by *S. uberis* in a time dependent manner and the alterations are not totally matched with those in the mammary glands.

### MECs Adopt Distinct Metabolism in Response to Various Microbial Stimuli Compared to Macrophages

**Figure 3:**
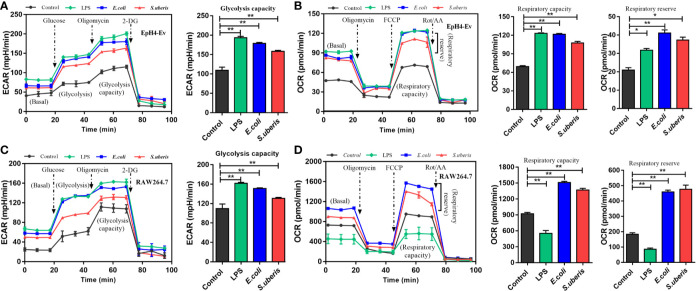
Metabolic responses in MECs and macrophages to various microbial stimuli. **(A, B)** Stimulation with 10 μg/mL LPS for 12 h or inactivated *S. uberis* or *E. coli* (MOI = 100) for 3 h at 37°C. Real-time changes in ECAR **(A)** and OCR **(B)** in EpH4-Ev cells were determined. **(C, D)** Stimulation with 100 ng/mL LPS for 12 h or inactivated *S. uberis* or *E. coli* (MOI = 5) for 3 h at 37°C. Real-time changes in ECAR **(C)** and OCR **(D)** in RAW264.7 cells were determined. Data are represented as mean ± SEM (n=3). *P < 0.05, **P < 0.01.

There are several cell types in mammary tissue including macrophages, polymorphonuclear neutrophilic leukocytes (PMN) and regulatory T cells (Treg). Metabolic reprogramming in most proinflammatory phenotype innate immune cells (i.e., dendritic cells, M1 macrophages, and natural killer (NK) cells) are characterized by elevated levels of TCA intermediates (succinate, citrate) and itaconate (an inflammation limited factor in proinflammatory phenotype cells inhibiting succinate dehydrogenase and causing increases of succinate and citrate) (33–35). Anti-inflammatory phenotype cells (i.e., M2 macrophages, natural killer T (NKT) cells and Treg cells) are associated with increased OXPHOS and decreased succinate and citrate (36–39). MECs involve in the occurrence and development of *S. uberis*-induced inflammation in our previous study (14, 16). EpH4-Ev cells incubated with *S. uberis* for 3 h have decreased citrate and α-ketoglutarate but increased itaconate levels (**Figure 2A** and **Supplementary Figure 6A**). We postulated that different metabolic patterns were present in MECs and proinflammatory phenotype innate immune cells challenged by *S. uberis*. Inactivated *S. uberis*, *Escherichia coli* (*E. coli*) and lipopolysaccharide (LPS) were used to stimulate EpH4-Ev cells and RAW 264.7 macrophages. LPS-stimulated RAW 264.7 macrophages developed an increase in ECAR (reflects glycolysis rate) and a decrease in OCR (reflects mitochondrial function and OXPHOS level) levels. MECs had an increase in ECAR and OCR levels after LPS challenge (**Figures 3A–D**). Both ECAR and OCR levels increased in the above 2 cell lines with inactivated *E. coli* and *S. uberis* stimulation (**Figures 3A–D**). These data indicate that MECs adopt distinct metabolism in response to various microbial stimuli compared to macrophages.

### Metabolic Reprogramming by Taurine in *S. uberis* Infection Coordinates With the Energy Supply and Production of Anabolic Intermediates

**Figure 4:**
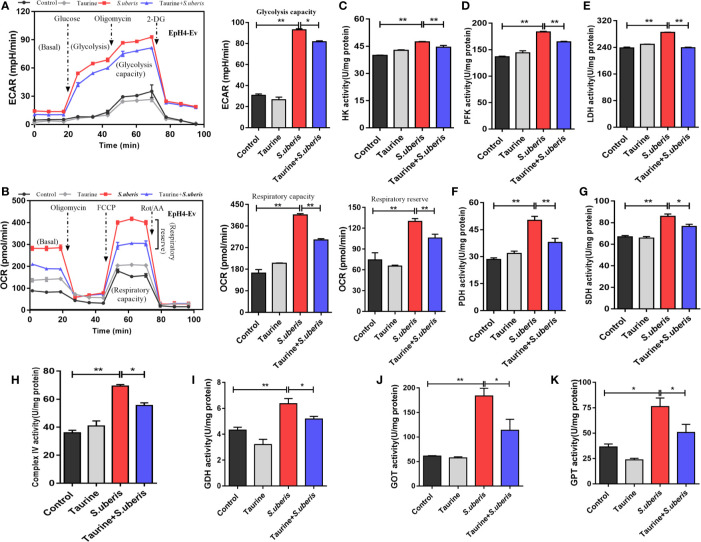
Taurine reprograms cellular metabolism by coordinaing the energy supply and anabolic intermediate production. **(A, B)** EpH4-Ev cells were pretreated with taurine for 24 h and then stimulated with inactivated *S. uberis* (MOI = 100) for 3 h at 37°C. Real-time changes in ECAR **(A)** and OCR **(B)** in EpH4-Ev cells were assessed during sequential treatment with oligomycin, carbonyl cyanide-4-(trifluoromethoxy) phenylhydrazone (FCCP), and antimycin A + rotenone. **(C–H)** After taurine pretreatment, EpH4-Ev cells were infected with *S. uberis* in mid-exponential phase (MOI = 10) for 3 h at 37°C. Enzyme activities related to glycolysis and OXPHOS were determined using commercial kits. **(I–K)** EpH4-Ev cells were pretreated with taurine for 24 h and then infected in mid-exponential phase for 3 h at 37°C with *S. uberis* (MOI = 10). The activities of enzymes linking amino acid and energy-related metabolites were determined using commercial kits. Data are represented as mean ± SEM (n = 3). *P < 0.05, **P < 0.01.

Cell metabolism generates energy and intermediates used to synthesize materials required to combat pathogen infection (40). Excessive or disordered mobilization results in cellular dysfunction. We evaluated whether taurine regulated metabolism associated with energy supply and anabolic intermediates production during *S. uberis* infection. Taurine pretreatment lowered ECAR and OCR levels during *S. uberis* challenge indicating that glycolysis and OXPHOS were inhibited in *S. uberis*-infected MECs (**Figures 4A, B**). Cells obtain energy and anabolic intermediates *via* biochemical reactive catalytic enzymes. Homeostasis is critical to cellular function and stability. We found that the activities of HK (the first and rate-limiting enzyme of glycolysis), PFK (the rate-limiting enzyme), and LDH increased in EpH4-Ev cells challenged with *S. uberis* (**Figures 4C–E**). The activities of PDH, SDH, and mitochondrial complex IV (key enzymes or components in OXPHOS) also elevated (**Figures 4F–H**). Taurine pretreatment significantly decreased these levels in infected EpH4-Ev cells (**Figures 4C–H**). These results agreed with real-time measurements of ECAR and OCR. Thus, MECs exhibit enhanced glycolysis and OXPHOS in response to *S. uberis* infection which are attenuated by taurine pretreatment.

To investigate whether *S. uberis* challenge promoted the conversion between amino acid and energy-related metabolites (such as glutamate and α-ketoglutarate) associated with a decrease in TCA intermediates, we assayed the activities of several key enzymes involved in energy and amino acid metabolism. *S. uberis* infection significantly increased GDH (**Figure 4I**), GOT (**Figure 4J**), and GPT activity (**Figure 4K**). Taurine pretreatment attenuated these changes. These results indicate that taurine reprograms *S. uberis*-induced metabolic changes to coordinate the energy supply and anabolic intermediate production.

### Taurine Balance Metabolism to Alleviate Inflammation Induced by *S. uberis*

*S. uberis* promotes proinflammatory mediator production in mammary glands and MECs (13, 16). EpH4-Ev cells were treated with 2-deoxy-D-glucose (2-DG; which inhibits glycolysis) and CPI-613 (which inhibits pyruvate dehydrogenase and α-ketoglutarate dehydrogenase, thereby blocking OXPHOS) to explore the relationship between energy metabolism and the inflammatory response. Key enzymes of glycolysis and OXPHOS detection showed that these 2 carbohydrate pathways were significantly restricted in EpH4-Ev cells with 2-DG (**Supplementary Figures 7A–C**) and CPI-613 (**Supplementary Figures 7D–F**), respectively. The effects of taurine pretreatment on glycolysis and OXPHOS were similar to those observed with 2-DG or CIP-613 treatment.

**Figure 5:**
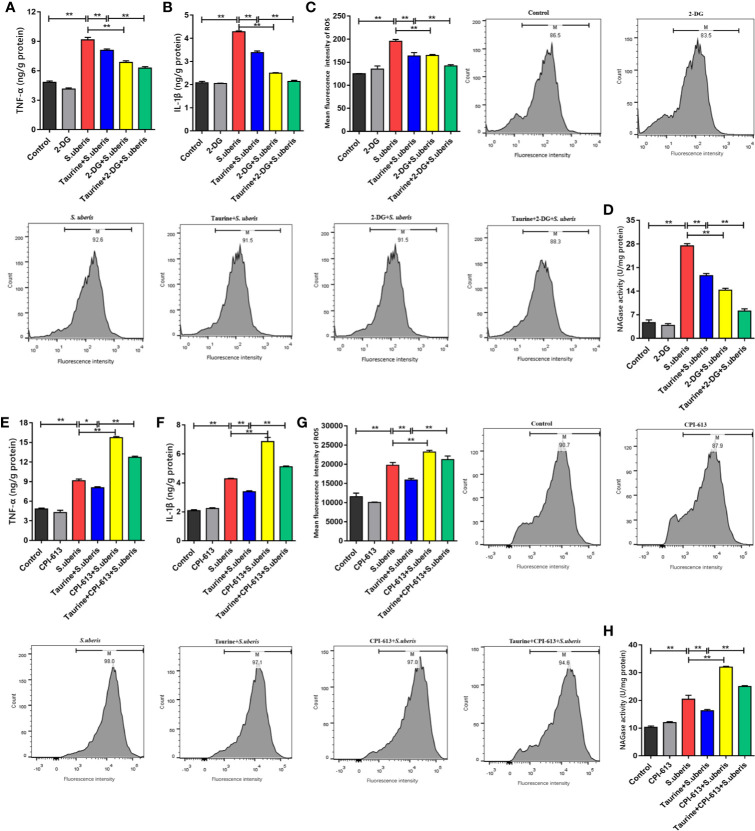
Taurine balance metabolism to alleviate inflammation induced by *S. uberis*. EpH4-Ev cells were pretreated with taurine for 24 h and infected with *S. uberis* in mid-exponential phase (MOI = 10) for 3 h at 37°C. The cells were treated with 5 mM 2-DG for 1 h to block glycolysis or with 25 μM CPI-613 for 12 h to block the TCA cycle prior to *S. uberis* infection. **(A, B, E, F)** TNF-α and IL-1β levels in the supernatants of EpH4-Ev cells pretreated with 2-DG **(A, B)** or CPI-613 **(E, F)** were measured using enzyme-linked immunosorbent assay (ELISA) kits. **(C, G)** Intracellular ROS content was evaluated by staining cells (10,000 cells/sample) with DCFH-DA, followed by analysis using CellQuest Pro acquisition and FlowJo software. **(D, H)** NAGase activity in the supernatants. Data are represented as mean ± SEM (n = 3). *P < 0.05, **P < 0.01.

Taurine pretreatment significantly decreased TNF-α and IL-1β levels in EpH4-Ev cells infected with *S. uberis* (**Figures 5A, B**), which was comparable to that of 2-DG treatment. Similarly, pretreatment of *S. uberis* infected EpH4-Ev cells with both 2-DG and taurine downregulated intracellular ROS levels more than pretreatment with taurine alone (**Figure 5C**). The supernatants of EpH4-Ev cells pretreated with taurine or glycolysis inhibitors and later infected with *S. uberis* had lower activity of NAGase (a marker of cell damage) than that of *S. uberis*-infected EpH4-Ev cells without pretreatment (**Figure 5D**). OXPHOS inhibition significantly increased TNF-α and IL-1β production (**Figures 5E, F**), contrary to taurine pretreatment. Moreover, CPI-613 treatment significantly increased intracellular ROS levels (**Figure 5G**) and NAGase activities (**Figure 5H**) in EpH4-Ev cells during *S. uberis* infection. Inhibited glycolysis with 2-DG diminished the inflammatory response while CPI-613 treatment results in a sharp increase of inflammatory mediators and resultant cell damage in *S. uberis* infection. These results suggest that glycolysis and OXPHOS work differently in MECs challenged with *S. uberis.* Taurine decreases both glycolysis and OXPHOS balancing whole energy metabolism in *S. uberis* infection, relieving inflammation and protecting cells from OXPHOS breakdown thus attenuating a proinflammatory metabolic reaction.

### Taurine Regulates Metabolic Alterations in *S. uberis* Infection by Inhibiting the mTOR Pathway

**Figure 6:**
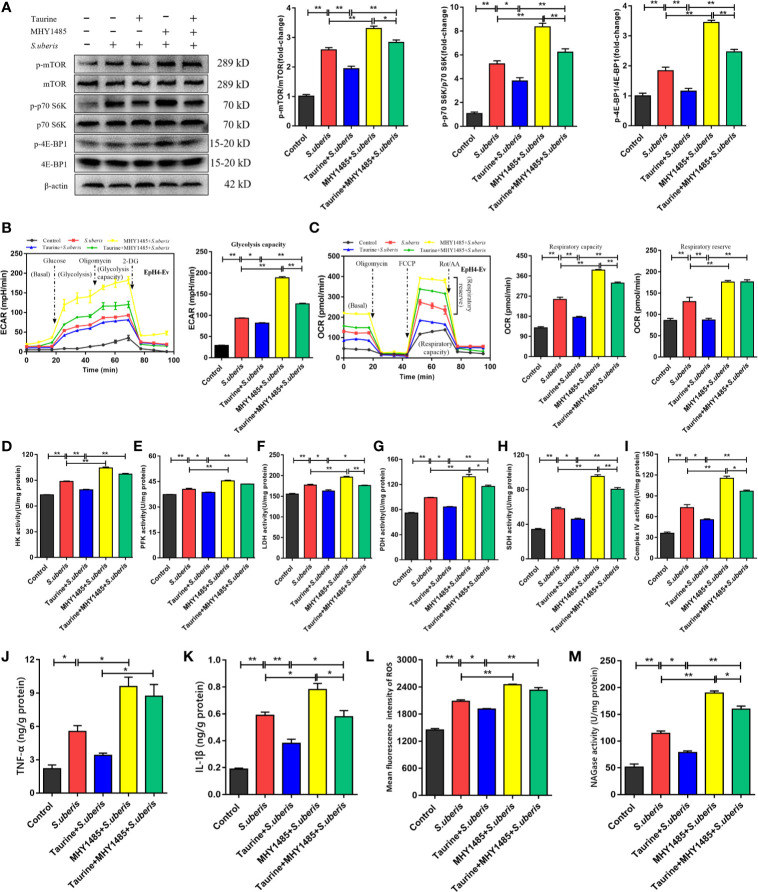
Taurine regulates metabolic alterations in *S. uberis* infection by inhibiting the mTOR pathway. **(A)** EpH4-Ev cells were pretreated with taurine for 24 h and infected with *S. uberis* in mid-exponential phase (MOI = 10) for 3 h at 37°C. The cells were pretreated with 100 nM MHY1485 (mTOR activator) for 24 h prior to *S. uberis* infection. The protein-expression levels of mTOR, p70 S6K, and 4E-BP1, as well as the levels of each phosphorylated protein (p-mTOR, p-p70 S6K, and p-4EBP1) were determined by western blotting. **(B, C)** EpH4-Ev cells were pretreated with taurine for 24 h and then stimulated with inactivated *S. uberis* (MOI = 100) for 3 h at 37°C. The cells were pretreated with 100 nM MHY1485 for 24 h before *S. uberis* infection. Real-time changes in the ECAR **(B)** and OCR **(C)** levels in EpH4-Ev cells were determined. **(D–I)** The relative activities of enzymes driving glycolysis and OXPHOS were determined using commercial kits. **(J, K)** TNF-α and IL-1β levels in EpH4-Ev cell supernatants pretreated with 100 nM MHY1485 were measured by ELISA. **(L)** Intracellular ROS contents were evaluated by staining cells (10,000/sample) with DCFH-DA, followed by analysis using CellQuest Pro acquisition and FlowJo software. **(M)** Supernatant NAGase activities were determined using commercial kits. Data are represented as mean ± SEM (n = 3). *P < 0.05, **P < 0.01.

Previously, we show that mTOR was involved in the production of various inflammatory mediators during *S. uberis* infection (16). Enhancement of the glycolytic pathway correlates with mTOR activity (40). Therefore, we hypothesized that taurine coupled with cellular metabolism through the mTOR pathway optimizes the production of inflammatory biomolecules. Taurine pretreatment inhibited mTOR pathway activity in EpH4-Ev cells during challenge with *S. uberis*, as indicated by decreased mTOR phosphorylation and decreased phosphorylation of its downstream targets, p70 S6K and 4E-BP1 (**Figure 6A**). The mTOR activator, MHY1485, reversed these changes in taurine-pretreated EpH4-Ev cells (**Figure 6A**). To verify whether mTOR mediated energy metabolism during *S. uberis* infection, we assessed ECAR and OCR levels using MHY1485. ECAR and OCR decreased in taurine-pretreated EpH4-Ev cells exposed to *S. uberis* (**Figures 6B, C**), whereas MHY1485 treatment increased these levels (**Figures 6B, C**). The activities of key glycolysis-associated (HK, PFK, LDH) and OXPHOS-associated (PDH, SDH, mitochondrial complex IV) enzymes or components were consistent with the changes of ECAR and OCR (**Figures 6D–I**). These findings confirm that taurine-mediated attenuation of metabolic responses in EpH4-Ev cells challenged with *S. uberis* is related to mTOR pathway activation. Additionally, TNF-α and IL-1β levels (**Figures 6J, K**), ROS production (**Figure 6L** and **Supplementary Figure 8**), and NAGase activity (**Figure 6M**) significantly decreased in taurine-pretreated EpH4-Ev cells during *S. uberis* infection, whereas MHY1485 treatment exerted the opposite effects of taurine. These data establish that the mTOR signaling pathway mediates metabolic alterations of taurine in *S. uberis* infection.

### Taurine Rescues mTOR-Mediated Metabolic Alterations in MECs *via* AMPK

**Figure 7:**
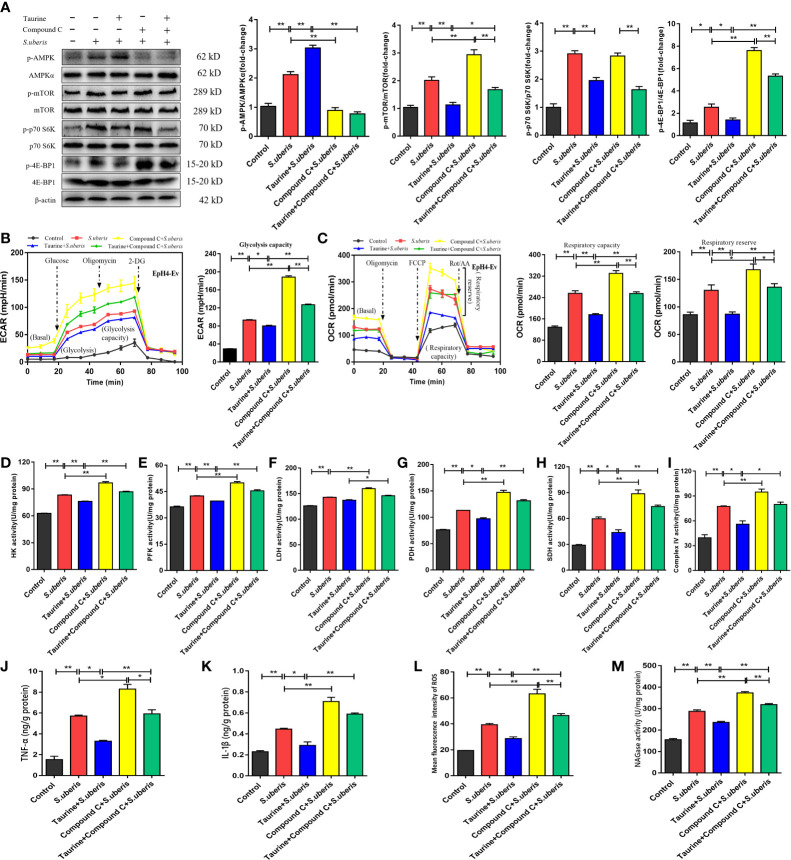
Taurine reverses mTOR-mediated metabolic alterations in MECs via AMPK. **(A)** EpH4-Ev cells were pretreated with taurine for 24 h and infected with *S. uberis* in mid-exponential phase (MOI = 10) for 3 h at 37°C. Cells were then further pretreated with 10 μM compound C (an AMPK inhibitor) for 1 h before *S. uberis* infection. The protein-expression levels of AMPKα, mTOR, p70 S6K, and 4E-BP1, and the corresponding phosphorylated proteins (p-AMPK, p-mTOR, p-p70 S6K, and p-4EBP1) were assayed by western blot. **(B, C)** EpH4-Ev cells were pretreated with taurine for 24 h and then with 10 μM Compound C prior to stimulation with inactivated *S. uberis* (MOI = 100) for 3 h at 37°C. Real-time changes in ECAR (B) and OCR (C) levels in EpH4-Ev cells were determined. **(D–I)** The relative activities of enzymes related to glycolysis and OXPHOS were determined using commercial kits. **(J, K)** TNF-α and IL-1β production in the supernatants of EpH4-Ev cells pretreated with 10 μM Compound C were measured via ELISA. **(L)** Intracellular ROS contents were evaluated by staining cells with DCFH-DA, followed by analysis (10,000 cells/sample) with CellQuest Pro acquisition and FlowJo software. **(M)** Supernatant NAGase activities were determined using commercial kits. Data are represented as mean ± SEM (n = 3). *P < 0.05, **P < 0.01.

As an AMPK activator (**Supplementary Figure 9A**), taurine has potential to negatively regulate mTOR activity resulting in coordination of cell metabolism with specific energy requirements (28). In EpH4-Ev cells, *S. uberis* infection increased the levels of phosphorylated AMPK versus controls (**Figure 7A**). To determine whether the AMPK pathway was involved in the metabolic recovery induced by taurine, we assessed the expression status of the AMPK-mTOR-p70 S6K-4E-BP1 pathway in MECs after taurine pretreatment. Immunoblot analyses showed that taurine pretreatment significantly increased AMPK phosphorylation levels and subsequently downregulated mTOR, p70 S6K, and 4E-BP1 phosphorylation all of which function immediately downstream of mTOR (**Figure 7A**). Pretreatment with the AMPK inhibitor, Compound C, blocked taurine-induced AMPK phosphorylation and reversed mTOR, p70 S6K, and 4E-BP1 phosphorylation (**Figure 7A**). ECAR and OCR levels increased, which concurred with the activity level of the mTOR pathway (**Figures 7B, C**). These data suggest that taurine-mediated regulation of metabolism in *S. uberis*-infected EpH4-Ev cells occurs *via* AMPK. Taurine significantly decreased HK, PFK, LDH, PDH, SDH, and mitochondrial complex IV activities in *S. uberis* infection, while pretreatment with Compound C reversed these taurine effects (**Figures 7D–I**). Taurine significantly decreased the levels of TNF-α and IL-1β (**Figures 7J, K**), ROS production (**Figure 7L** and **Supplementary Figure 9B**), and NAGase activity (**Figure 7M**) in *S. uberis*-infected EpH4-Ev cells, whereas Compound C pretreatment reversed these taurine-induced effects.

## Discussion

Mastitis remains an ongoing health problem, especially when caused by persistent intracellular bacterial infection, such as *S. uberis* (2, 8). Recent findings in our lab have shown that control of inflammation protects host from excessive or chronic damage induced by *S. uberis* challenge (14). We have found that taurine plays an important role in *S. uberis* infection for protecting host from inflammation (12–14). As metabolism and inflammation are closely interconnected, we investigated whether taurine ameliorates *S. uberis*-induced mastitis *via* metabolic alteration. We employed GC-TOF-MS non-targeted metabolomics methodology to analyze the metabolic changes in mammary glands and MECs after *S. uberis* infection. The resultant data demonstrate that during *S. uberis* invasion, the intermediates of glycolysis, the pentose phosphate pathway, lipid metabolism and amino acid metabolism are significantly increased while the concentration of TCA cycle intermediates decrease. In addition, taurine has a regulatory effect on these changes, which suggests that its regulation of *S. uberis* infection is related to metabolism. The metabonomic data presented here show that the alteration of metabolic pattern in MECs are not completely matched with those in mammary tissue. Mammary glands are complex and contain many cell types besides MECs partially obfuscating *in vivo* comparisons.

MECs are the major functional cells of the mammary gland, and although they are not professional immune cells, they are involved in inflammatory responses to *S. uberis* infection (14, 16). The role of MECs in metabolic changes occurring in the mammary gland, and the significance of these metabolic changes are not known. It has been reported that metabolic changes impact cell functions (41), and metabolic reprogramming is associated with an adjustment of host defense ability (42). For example, during activation, immune cells, such as macrophages, monocytes, and DCs, modulate their metabolism by increasing glycolytic fluxes enhancing phagocytic capacity or secretion of immunoregulatory cytokines (36). Human monocytes stimulated with LPS shift from OXPHOS to glycolysis, thereby activating host defense factors production (43). Further, evidences have demonstrated that *Citrobacter rodentium* infection caused intestinal epithelial cells metabolic rewiring toward aerobic glycolysis (44). Elevated glycolysis is also closely associated with innate immunity of epithelial cells, like *Staphylococcus aureus*-challenged human keratinocytes (45). In the current study, the levels of glycolysis and OXPHOS in MECs increased during *S. uberis* infection. By means of these metabolic alerts, the increased energy and intermediary requirements were met for defensive host immune responses (46). This indicates that metabolic reprogramming of MECs plays a crucial role in mammary defense.

Different from typical proinflammatory phenotypes, immune cells (M1 macrophages, NKs and PMN, etc.) and even intestinal epithelial cells (37, 44, 47, 48), MECs undergo a unique metabolic reprogramming pattern characterized by decreased production of TCA cycle intermediates accompanied by activated OXPHOS. This was further confirmed by the activities of key enzymes in glycometabolism. Specific metabolic alterations in cells accommodate distinct functional outputs. Glycolysis upregulation is more likely to occur under inflammatory conditions, whereas OXPHOS represents upregulation of anti-inflammatory pathways (39, 49–51). Increased activity of the glycolytic pathway likely promotes inflammation and resistance to *S. uberis* infection, although the precise reason for OXPHOS is also upregulated in *S. uberis*-infected MECs remains unclear. A likely explanation is that MECs act as nonprofessional immune cells and are, therefore, not equipped to counter the acute challenge during *S. uberis* infection. Therefore, they may enhance OXPHOS by producing more energy and contributing more intermediates to constrain infection and subsequent inflammation, thereby reducing cell damage (46). Our results show that glycolysis inhibition in MECs through 2-DG reduced ROS, TNF-α, and IL-1β secretion alleviates inflammation and subsequent injury. Blocking OXPHOS through CPI-613 leads to increased inflammation. The unique metabolic shift in MECs may represent a specific self-protective mechanism that relieves excessive inflammation during *S. uberis* infection.

In mammalian cells, taurine is derived from cysteine and has many biological roles, such as conjugation of bile acids (absorption and transport of lipids), osmoregulation, and regulation of glucose and lipid homeostasis (energy metabolism) (20, 52). In diabetic rats, taurine supplementation reduces abdominal body fat while improving glucose tolerance (53). In the current *in vitro* metabonomic study, taurine reduces the activities of several key glycolytic enzymes, the TCA cycle and amino-acid metabolism, suggesting that taurine alleviates excessively mobilized cellular metabolite and affects energy metabolism in *S. uberis* activated MECs.

The AMPK-mTOR pathway functions as a signaling nexus for regulating cellular metabolism, energy homeostasis, and cell growth under various nutrient stress. In most cells, AMPK plays an important role in sensing energy status and restoring energy balance (28). We find taurine is an AMPK activator in this study. AMPK activators exert anti-inflammatory effects by directly activating catabolism and inhibiting anabolism (39). Our data show that taurine constrained both glycolysis and OXPHOS, indicating that it may regulate overall energy production in *S. uberis* infected MECs. We conclude that AMPK mediates metabolism regulation in *S. uberis* infected MECs. mTOR is a central controller of cell growth and proliferation through its influence on metabolism, especially on protein synthesis. AMPK is a master regulator of metabolism that acts upstream of mTOR. Its negative control of the mTOR pathway correlates with glycolytic activity. In macrophages, mTOR-mediated proinflammatory signal stimulation can drive mTOR-dependent metabolic-flux reprogramming (40). Additionally, LPS-treated macrophages display increased inflammatory responses through PI3K/mTOR pathway activation (54). These data are consistent with our previous finding that PI3K/Akt/mTOR-pathway activation promotes inflammation in *S. uberis*-infected MECs (16). It is therefore possible that taurine regulates metabolism through the AMPK-mTOR pathway thus reducing inflammation.

In the current study, taurine alleviates *S. uberis* induced-inflammation by activating AMPK which inhibits the mTOR pathway, resulting in decreased proinflammatory cytokine production and decreased anabolic and catabolic processes. Treatment with AMPK inhibitors or mTOR agonists considerably increases the expression of inflammatory factors and ROS, causing further cell damage. Compound C, an AMPK inhibitor, reverses taurine-induced AMPK phosphorylation and altered mTOR activity thus regulating the corresponding metabolic changes and anti-inflammatory responses. Taken together, our results show that taurine decreases overall energy metabolism in the host by regulating the AMPK-mTOR pathway, reducing inflammation caused by *S. uberis* infection.

Our findings show that metabolic reprogramming drives *S. uberis*-induced inflammation in mammary glands and MECs, and that taurine abrogates this metabolic reprogramming and dysfunction, thereby reducing inflammation. Taurine effectively inhibits overall energy metabolism by boosting AMPK levels reducing the activity of the mTOR pathway, which markedly reduces *S. uberis*-induced inflammatory responses in MECs. Our findings shed light on the close relationship between cellular-metabolic pathways and inflammation of mammary glands and MECs. This study indicates that amelioration of bovine mammary epithelial cell metabolism is an effective way to prevent bacterial mastitis in dairy cows.

## Data Availability Statement

The original contributions presented in the study are included in the article/**Supplementary Material**. Further inquiries can be directed to the corresponding author.

## Ethics Statement

The animal study was reviewed and approved by the committee on the Use and Care of Animals of Nanjing Agricultural University (Nanjing, China).

## Author Contributions

RL and ZXW contributed equally as co-first authors. RL and ZXW designed and performed the experiments and analyzed the data. ZXW wrote the manuscript. RL performed the metabonomics experiments and analyzed the data. YX and ZLW performed the GC–TOF-MS and metabolomics analyses. YZ assisted with the *in vivo* infection trial and ROS measurements. SF assisted in experiments related to extracellular flux analysis. XL aided in the detection of relative enzyme activities and in the ELISAs. XH provided advice for the *in vivo* studies. ZL and YY provided guidance and advice. YX provided advice and reagents and oversaw a portion of the work. JM conceived ideas and oversaw the research program. All authors contributed to the article and approved the submitted version.

## Funding

This project was supported by grants from the National Natural Science Foundation of China (No. 32072867, 31672515), the Key Project of Inter-governmental International Scientific and Technological Innovation Cooperation (No.2018YFE0102200), and the Project Funded by the Priority Academic Program Development of Jiangsu Higher Education Institutions.

## Conflict of Interest

The authors declare that the research was conducted in the absence of any commercial or financial relationships that could be construed as a potential conflict of interest.
