# Supplementary material for: Taurine Reprograms Mammary-Gland Metabolism and Alleviates Inflammation Induced by Streptococcus uberis in Mice
Source: Front Immunol. 2021 Jun 10;12:696101. doi: 10.3389/fimmu.2021.696101 (PMC8222520; doi:10.3389/fimmu.2021.696101)
Supplement: Supplementary file 1 [file DataSheet_1.zip › Supplementary materials-clean line numbers/Supplementary materials-clean line numbers.pdf]

## Supplementary Figures

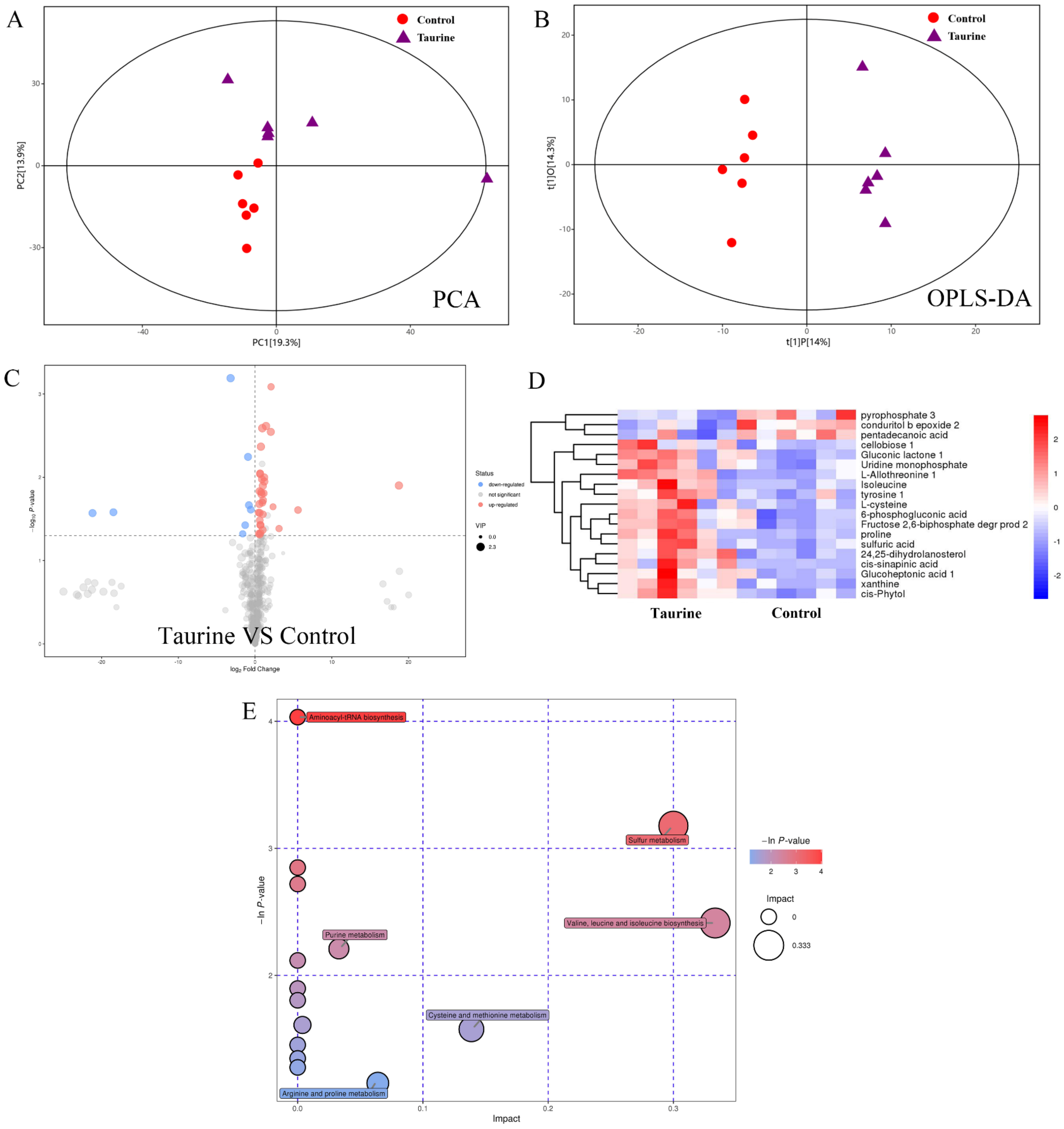


**Figure S1. Metabolic profile in mammary glands of taurine-administrated C57BL/6J mouse.** Pregnant C57BL/6J (B6) mice were administered 100 mg/kg taurine (dissolved in sterile progeny-free saline) or an equal volume of saline by gavage daily until parturition. At 72 h after parturition, mice from the Taurine group were infused

with 50 μL sterile progeny-free saline into the L4 and R4 teats. At 24 h post of infusion, mammary glands were collected for GC–TOF-MS analysis. 6 mice of each group were sampled (n=6). **(A)** PCA score map. **(B)** Orthogonal partial least squares discriminant analysis (OPLS-DA) score plot. **(C)** Volcano plot derived from the different metabolite profiles of the Taurine group versus the control group. **(D)** Significant changes in metabolites in C57BL/6J mice mammary glands (Taurine group versus control group) are shown in the heatmap. **(E)** Metabolome map of significant metabolic pathways of mammary gland metabolites (Taurine group versus control group). The x-axis represents pathway enrichment, and the y-axis represents the pathway impact. Large sizes and dark colors represent major pathway-enrichment and high pathway-impact values, respectively.

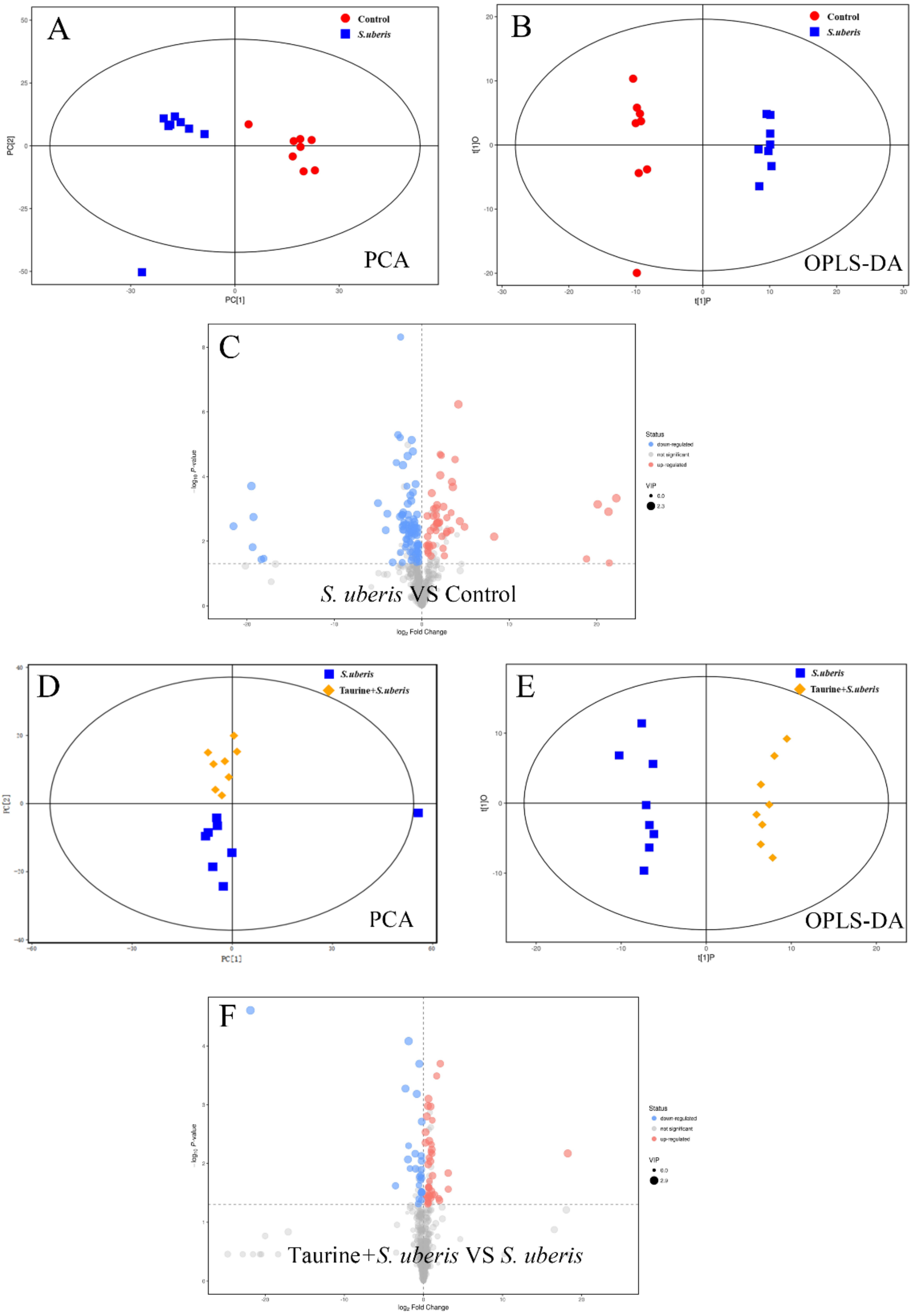

A
Control
S.uberis
PCA
PC[1]
PC[2]
B
Control
S.uberis
OPLS-DA
t[1]P
t[1]O
C
Status
down-regulated
not significant
up-regulated
VIP
S. uberis VS Control
log2 Fold Change
-log10 P-value
D
S.uberis
Taurine+S.uberis
PCA
PC[1]
PC[2]
E
S.uberis
Taurine+S.uberis
OPLS-DA
t[1]P
t[1]O
F
Status
down-regulated
not significant
up-regulated
VIP
Taurine+S. uberis VS S. uberis
log2 Fold Change
-log10 P-value

**Figure S2. Global-metabolite profiling of taurine-dependent regulation of metabolic homeostasis in C57BL/6J mouse mammary glands during *S. uberis* infection.**

Pregnant C57BL/6J (B6) mice were administered 100 mg/kg taurine (dissolved in sterile pyrogen-free saline) or an equal volume of saline by gavage daily until parturition. At 72 h after parturition, mice from the *S. uberis* group and Taurine + *S. uberis* group were infused with 100 CFU *S. uberis* in 50 μL into the L4 and R4 teats. At 24 h PI, mammary glands were collected for GC–TOF-MS analysis. 8 mice of each group were sampled (n=8). **(A–C)** Global-metabolite profiling of the *S. uberis* group versus the control group. **(A)** PCA score map, **(B)** Orthogonal partial least squares discriminant analysis (OPLS-DA) score plot, and **(C)** volcano plot derived from the different metabolite profiles of the *S. uberis* group versus the control group. **(D–F)** Global-metabolite profiles of the Taurine + *S. uberis* group versus the *S. uberis* group. **(D)** PCA score map, **(E)** OPLS-DA score plot, and **(F)** volcano plot derived from the different metabolite profiles of the Taurine + *S. uberis* group versus the *S. uberis* group. Each group contained 8 mice. The red dots in the volcano plot indicate different metabolites that were more abundant in the comparator group, whereas the blue dots represent lower concentrations. Fold-changes refer to the mean peak area of the first group/mean peak area of the second group.

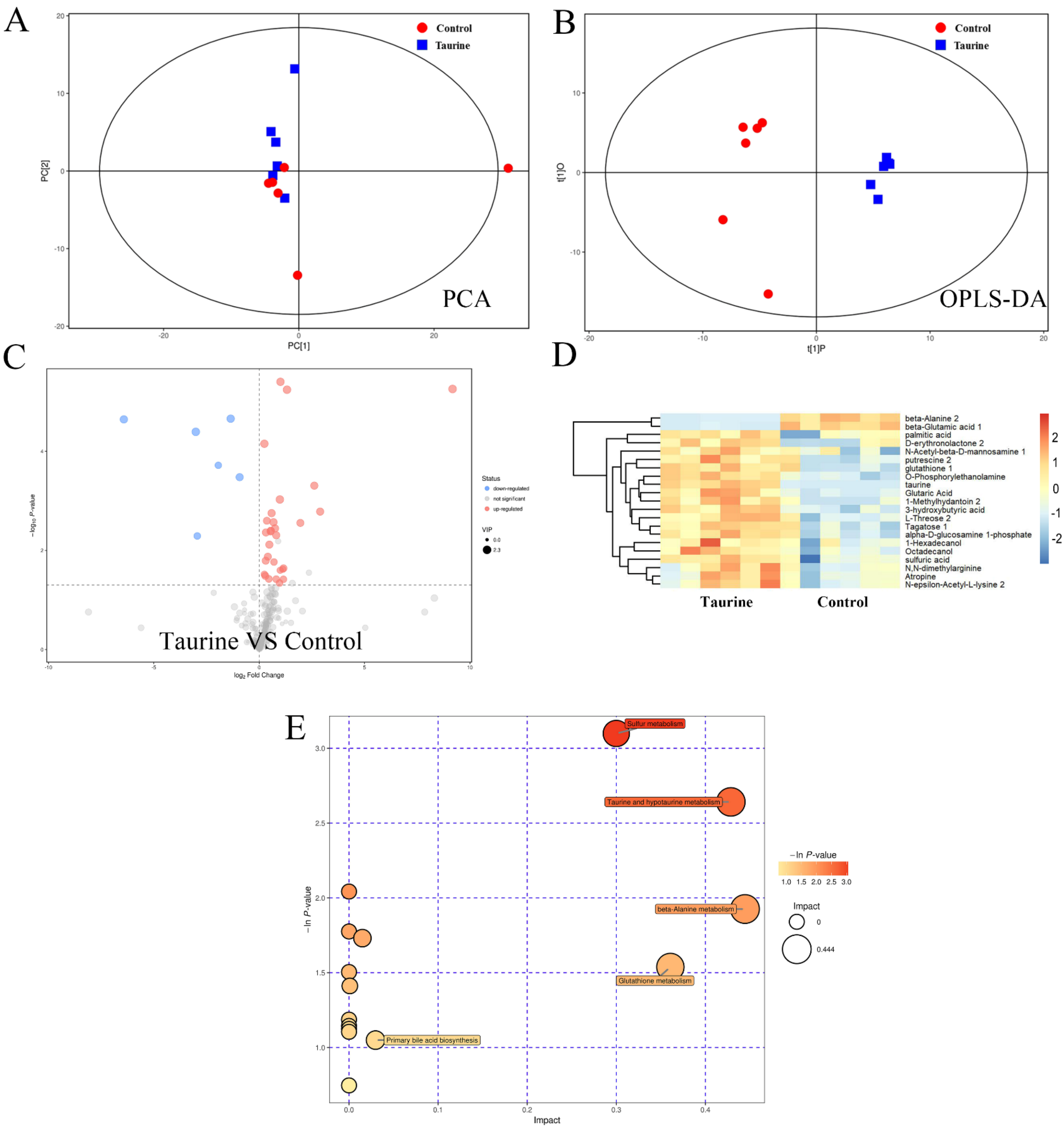


**Figure S3. Metabolic profile in taurine-administrated EpH4-Ev cells.**
EpH4-Ev cells were pretreated with taurine for 24 h and then cellular metabolites were extracted and detected by GC–TOF-MS. Six samples of each group were processed (n=6). **(A)** PCA score map. **(B)** OPLS-DA score plot. **(C)** Volcano plot derived from the different metabolite profiles of the Taurine group versus the control group. **(D)** Significant changes in metabolites in EpH4-Ev cells (Taurine group versus control

group) are shown in the heatmap. **(E)** Metabolome map of significant metabolic pathways of mammary gland metabolites (Taurine group versus control group). The x-axis represents pathway enrichment, and the y-axis represents the pathway impact. Large sizes and dark colors represent major pathway-enrichment and high pathway-impact values, respectively.

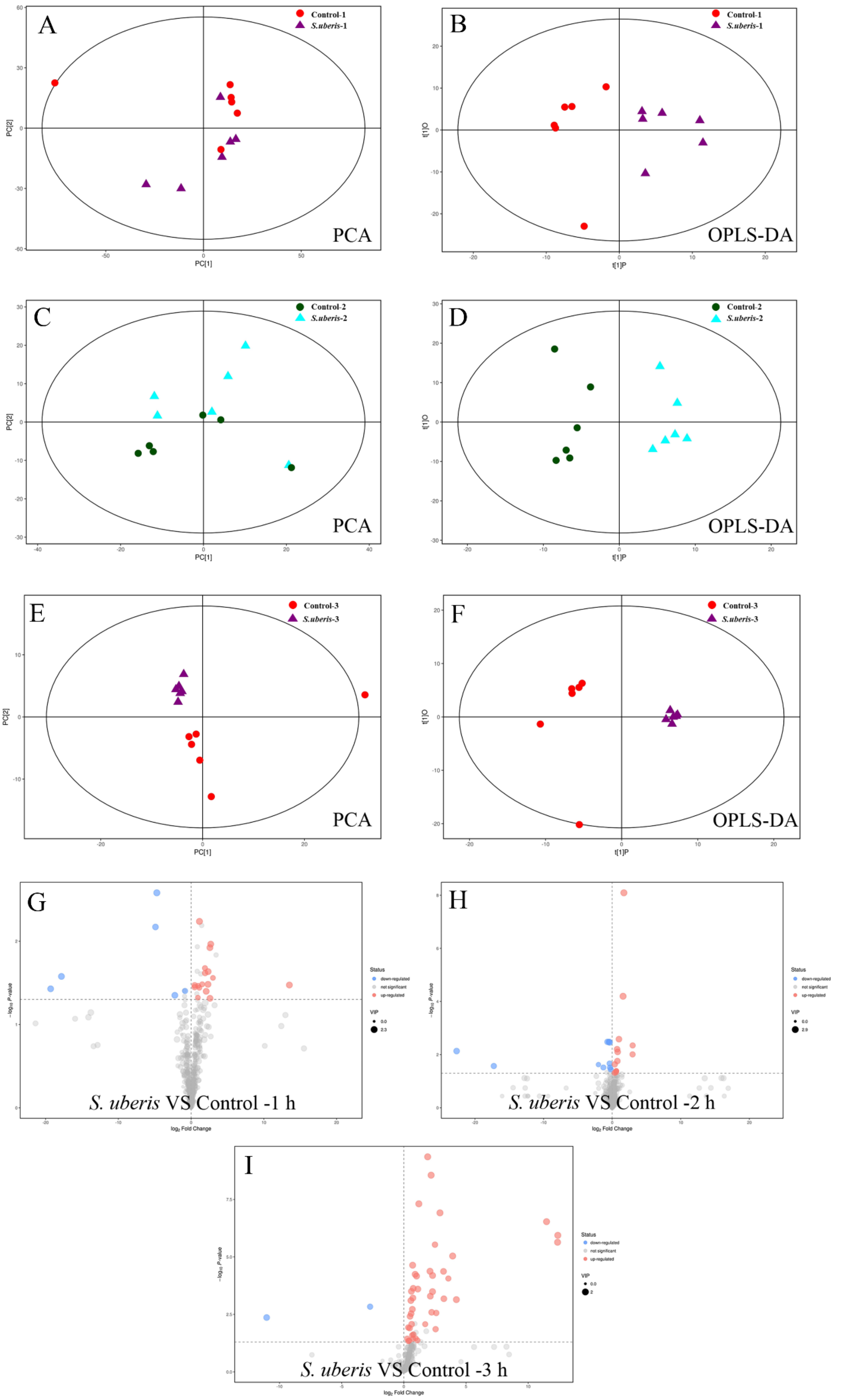
A
Control-1
S.uberis-1
PCA
PC[1]
PC[2]
B
Control-1
S.uberis-1
OPLS-DA
t[1]P
t[1]O
C
Control-2
S.uberis-2
PCA
PC[1]
PC[2]
D
Control-2
S.uberis-2
OPLS-DA
t[1]P
t[1]O
E
Control-3
S.uberis-3
PCA
PC[1]
PC[2]
F
Control-3
S.uberis-3
OPLS-DA
t[1]P
t[1]O
G
S. uberis VS Control -1 h
H
S. uberis VS Control -2 h
I
S. uberis VS Control -3 h
Status
down-regulated
not significant
up-regulated
VIP
log2 Fold Change
-log10 P-value

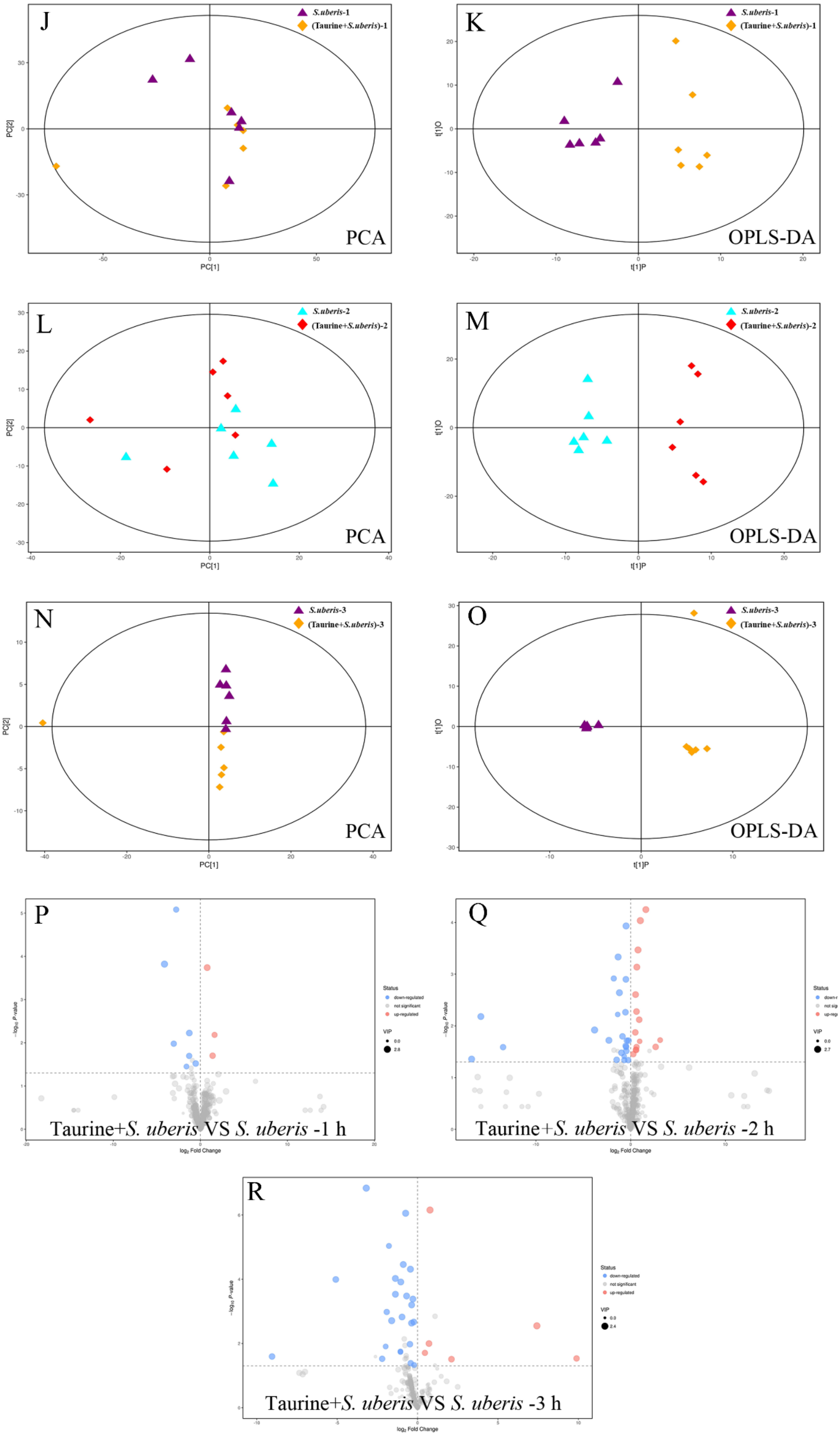

J
S.uberis-1
(Taurine+S.uberis)-1
PC[1]
PC[2]
PCA
K
S.uberis-1
(Taurine+S.uberis)-1
t[1]P
t[1]O
OPLS-DA
L
S.uberis-2
(Taurine+S.uberis)-2
PC[1]
PC[2]
PCA
M
S.uberis-2
(Taurine+S.uberis)-2
t[1]P
t[1]O
OPLS-DA
N
S.uberis-3
(Taurine+S.uberis)-3
PC[1]
PC[2]
PCA
O
S.uberis-3
(Taurine+S.uberis)-3
t[1]P
t[1]O
OPLS-DA
P
Taurine+S. uberis VS S. uberis -1 h
Status
down-regulated
not significant
up-regulated
VIP
log2 Fold Change
Q
Taurine+S. uberis VS S. uberis -2 h
Status
VIP
log2 Fold Change
R
Taurine+S. uberis VS S. uberis -3 h
Status
down-regulated
not significant
up-regulated
VIP
log2 Fold Change

**Figure S4. Global-metabolite profiling of taurine-dependent regulation of metabolic homeostasis in EpH4-Ev cells challenged with *S. uberis* at different times.**

EpH4-Ev cells were pretreated with taurine for 24 h and then infected with *S. uberis* in mid-exponential phase (MOI = 10) for 1, 2, or 3 h at 37°C. The cellular metabolites were extracted and detected by GC–TOF-MS. Six samples of each group were processed (n=6). **(A–I)** Global-metabolite profiling of the *S. uberis* group versus the control group, following different times of *S. uberis* infection. PCA score map (A, B, C), OPLS-DA score plot (D, E, F), and volcano plot (G, H, I) derived from the different metabolite profiles of the *S. uberis* group versus the control group following *S. uberis* infection for 1, 2, or 3 h. **(J–R)** Global-metabolite profiling of the Taurine + *S. uberis* group versus the control group after *S. uberis* infection for different times. PCA score map (J, K, L), OPLS-DA score plot (M, N, O), and volcano plot (P, Q, R) derived from the different metabolite profiles of the Taurine +*S. uberis* group versus the *S. uberis* group after *S. uberis* infection for 1, 2, or 3 h. Six samples were included in each group. The red dots in the volcano plot indicate different metabolites that were more abundant in the comparative group, whereas the blue dots represent lower concentrations. Fold-changes refer to the mean peak area of the first group/mean peak area of the second group.

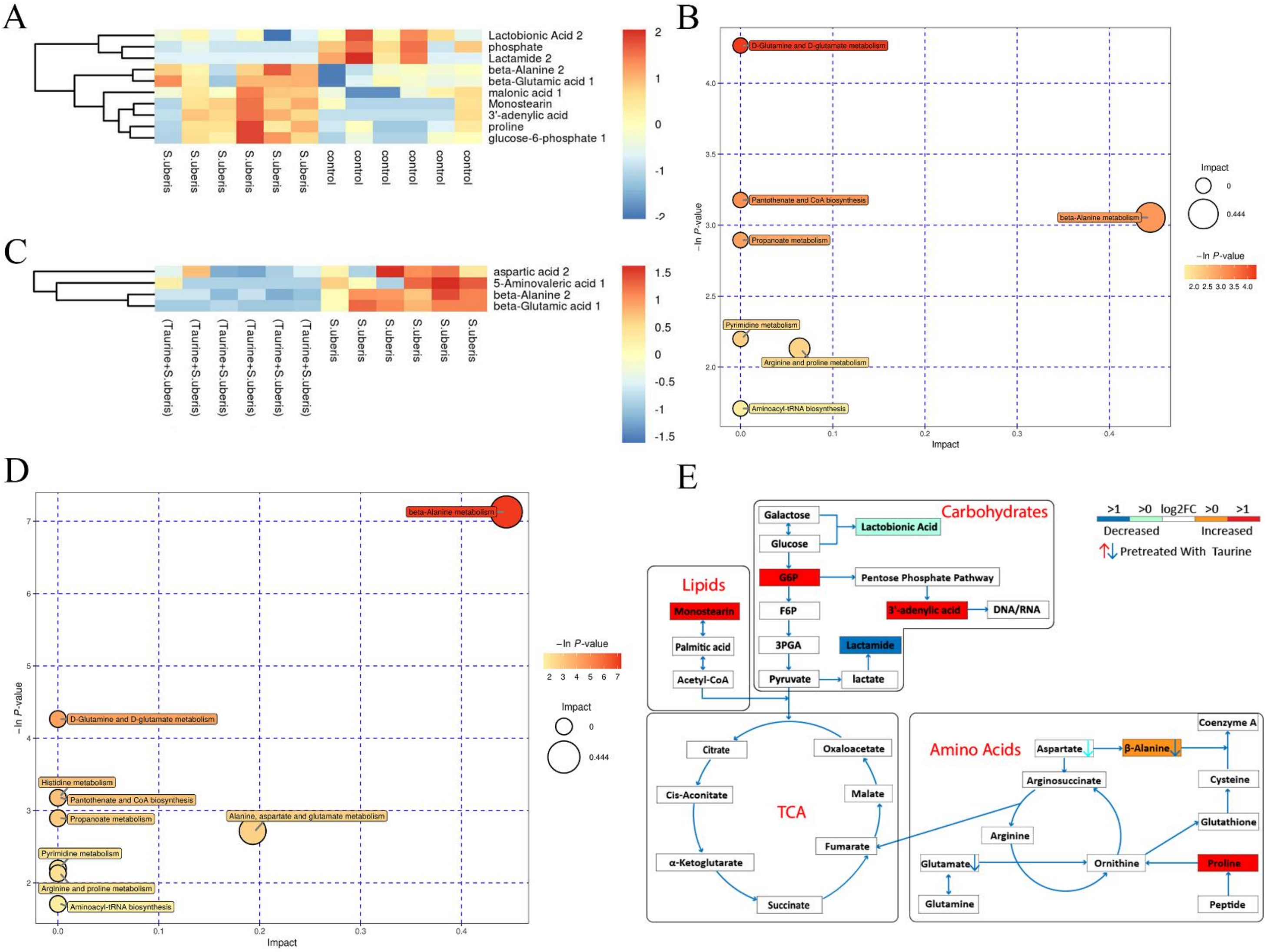


**Figure S5. Taurine pretreatment lowers the metabolic level in EpH4-Ev cells during a 1-h *S. uberis* infection.**

EpH4-Ev cells were pretreated with taurine for 24 h and then infected with *S. uberis* in mid-exponential phase (MOI = 10) for 1 h at 37°C. Cellular metabolites were extracted and detected by GC–TOF-MS. **(A)** Significant fold-changes in metabolites in EpH4-Ev cells are represented in a heatmap depicting changes in the *S. uberis* group versus the control group. **(B)** Metabolome map of significant metabolic pathways characterized in terms of metabolites in EpH4-Ev cells for the *S. uberis* group versus the control group. The x-axis represents pathway enrichment, and the y-axis represents pathway impact. Large sizes and dark colors represent major pathway-enrichment and high pathway-impact values, respectively. **(C)** Significant fold-changes in metabolites in EpH4-Ev cells are represented in a heatmap depicting changes in the Taurine + *S. uberis* group versus the *S. uberis* group. **(D)** Metabolome map of significant metabolic pathways characterized in terms of metabolites in EpH4-Ev cells of the Taurine + *S. uberis* group versus the *S. uberis* group. **(E)** Model of the metabolomics responses to *S. uberis* infection for 1 h after taurine pretreatment for 24 h in EpH4-Ev cells.

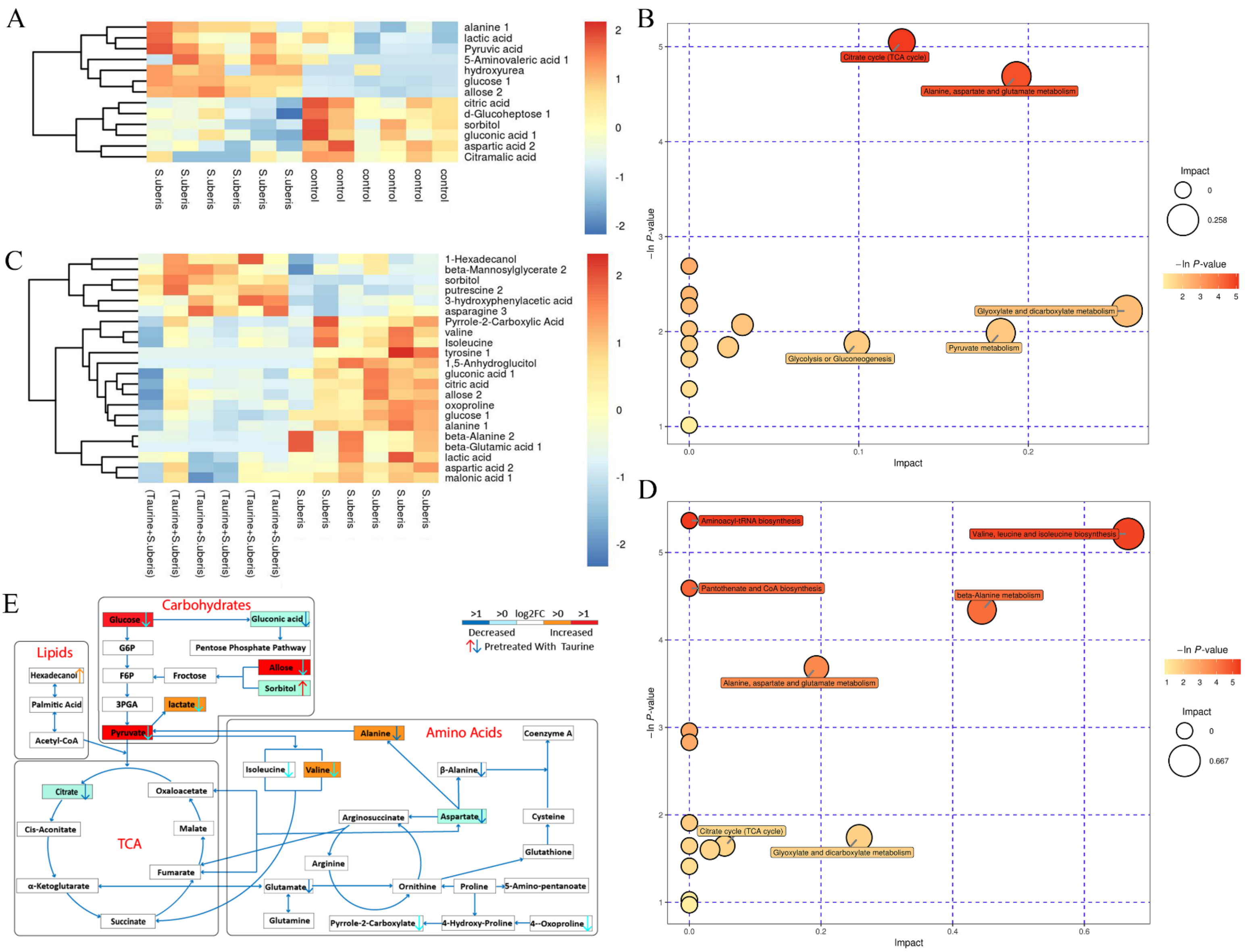

A
alanine 1
lactic acid
Pyruvic acid
5-Aminovaleric acid 1
hydroxyurea
glucose 1
allose 2
citric acid
d-Glucoheptose 1
sorbitol
gluconic acid 1
aspartic acid 2
Citramalic acid
S.uberis
control
2
1
0
-1
-2
B
Citrate cycle (TCA cycle)
Alanine, aspartate and glutamate metabolism
Glyoxylate and dicarboxylate metabolism
Pyruvate metabolism
Glycolysis or Gluconeogenesis
−ln P-value
Impact
0.258
C
1-Hexadecanol
beta-Mannosylglycerate 2
sorbitol
putrescine 2
3-hydroxyphenylacetic acid
asparagine 3
Pyrrole-2-Carboxylic Acid
valine
Isoleucine
tyrosine 1
1,5-Anhydroglucitol
gluconic acid 1
citric acid
allose 2
oxoproline
glucose 1
alanine 1
beta-Alanine 2
beta-Glutamic acid 1
lactic acid
aspartic acid 2
malonic acid 1
(Taurine+S.uberis)
S.uberis
D
Aminoacyl-tRNA biosynthesis
Valine, leucine and isoleucine biosynthesis
Pantothenate and CoA biosynthesis
beta-Alanine metabolism
Alanine, aspartate and glutamate metabolism
Citrate cycle (TCA cycle)
Glyoxylate and dicarboxylate metabolism
0.667
E
Carbohydrates
Glucose
Gluconic acid
G6P
Pentose Phosphate Pathway
F6P
Froctose
Allose
Sorbitol
3PGA
lactate
Pyruvate
Lipids
Hexadecanol
Palmitic Acid
Acetyl-CoA
>1 >0 log2FC >0 >1
Decreased Increased
Pretreated With Taurine
Amino Acids
Alanine
Coenzyme A
Isoleucine
Valine
β-Alanine
Arginosuccinate
Aspartate
Cysteine
Arginine
Glutathione
Glutamate
Ornithine
Proline
5-Amino-pentanoate
Glutamine
Pyrrole-2-Carboxylate
4-Hydroxy-Proline
4--Oxoproline
TCA
Citrate
Oxaloacetate
Cis-Aconitate
Malate
Fumarate
α-Ketoglutarate
Succinate

**Figure S6. Taurine decreases metabolic disturbances caused by *S. uberis* infection for 2 h *in vitro*.** EpH4-Ev cells were pretreated with taurine for 24 h and then infected with *S. uberis* in mid-exponential phase (MOI = 10) for 2 h at 37°C. Cellular metabolites were extracted and detected by GC–TOF-MS. **(A)** Significant fold-changes in metabolites in EpH4-Ev cells are represented in a heatmap depicting changes in the *S. uberis* group versus the control group. **(B)** Metabolome map of significant metabolic pathways characterized in terms of metabolites in EpH4-Ev cells of the *S. uberis* group versus the control group. The x-axis represents pathway enrichment, and the y-axis represents pathway impact. Large sizes and dark colors represent major pathway-enrichment and high pathway-impact values, respectively. **(C)** Significant fold-changes in metabolites in EpH4-Ev cells are represented in a heatmap depicting changes in the Taurine + *S. uberis* group versus the *S. uberis* group. **(D)** Metabolome map of significant metabolic pathways characterized in terms of metabolites in EpH4-Ev cells in the Taurine + *S. uberis* group versus the *S. uberis* group. **(E)** Model of the metabolomics response to *S. uberis* infection for 2 h after taurine pretreatment for 24 h in EpH4-Ev cells.

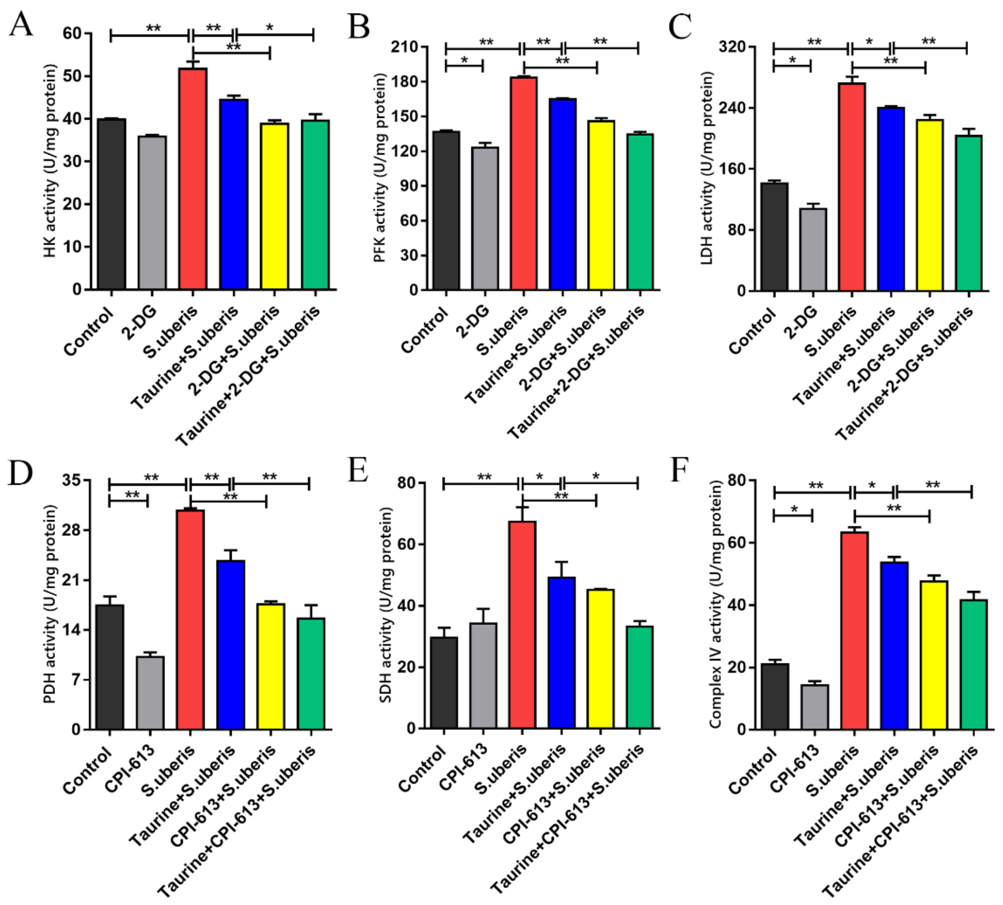


**Figure S7. The glycolysis inhibitor, 2-DG and the OXPHOS (TCA cycle) inhibitor, CPI-613 blocked energy metabolism in EpH4-Ev cells.**

**(A–C)** EpH4-Ev cells were pretreated with taurine for 24 h and then infected with *S. uberis* in mid-exponential phase (MOI = 10) for 3 h at 37°C. Cells were treated with 5 mM 2-DG for 1 h to block glycolysis before *S. uberis* infection. The relative activities of enzymes associated with glycolysis were determined. **(D–F)** EpH4-Ev cells were pretreated with taurine for 24 h and then infected with *S. uberis* in mid-exponential phase (MOI = 10) for 3 h at 37°C. Cells were treated with 25 μM CPI-613 for 12 h to block the TCA cycle before *S. uberis* infection. The relative activities of enzymes associated with OXPHOS were determined. Data are represented as mean ± SEM (n=3). $*P < 0.05$, $**P < 0.01$.

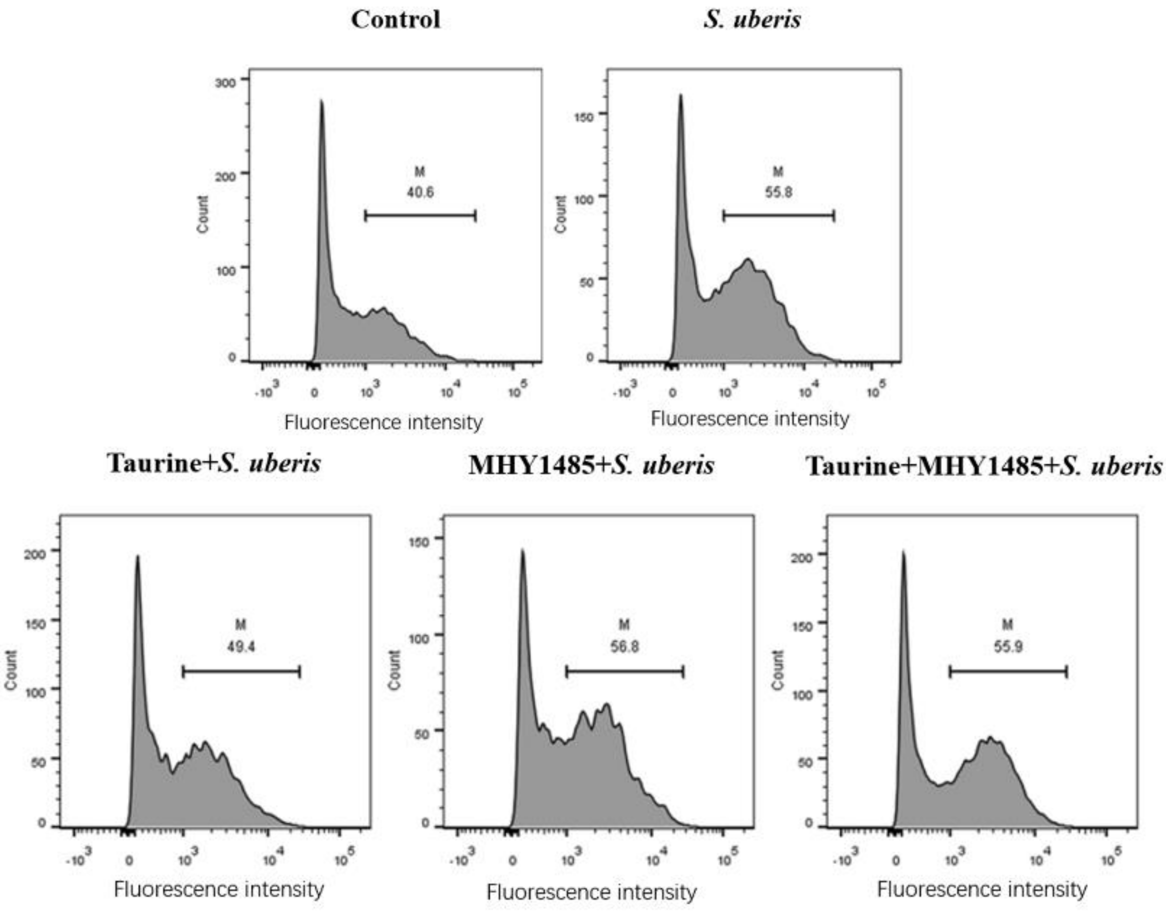


**Figure S8. Taurine regulated ROS production induced by *S. uberis* infection in MECs via the mTOR pathway.**

EpH4-Ev cells were pretreated with taurine for 24 h and then infected with *S. uberis* in mid-exponential phase (MOI = 10) for 3 h at 37°C. Cells were pretreated with 100 nM MHY1485 (an mTOR activator) for 24 h before *S. uberis* infection. Intracellular ROS contents were evaluated by staining cells (10,000/sample) with DCFH-DA, followed by analysis with CellQuest Pro acquisition software and FlowJo software. Data are represented as mean ± SEM (n=3). *$P < 0.05$, **$P < 0.01$.

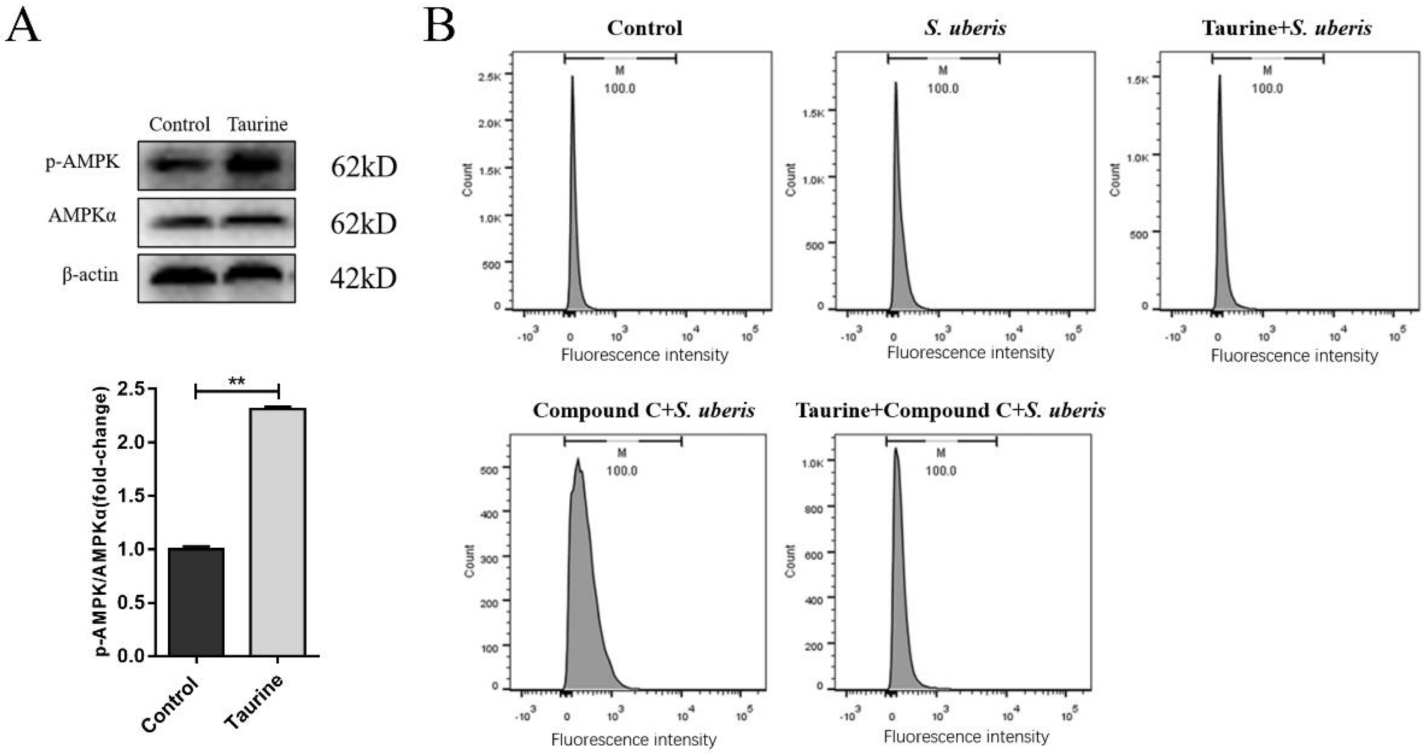


**Figure S9. Taurine acts as an AMPK activator and regulates ROS production induced by *S. uberis* infection via AMPK-mTOR signaling.**

**(A)** EpH4-Ev cells were pretreated with taurine for 24 h and then infected with *S. uberis* in mid-exponential phase (MOI = 10) for 3 h at 37°C. Cells were pretreated with Compound C (10 μM) for 1 h before *S. uberis* infection. The protein-expression level of AMPKα and its phosphorylation level (p-AMPK) in uninfected cells were determined by western blot analysis. **(B)** Intracellular ROS contents were evaluated by staining cells (10,000/sample) with DCFH-DA, followed by analysis with CellQuest Pro acquisition software and FlowJo software. Data are represented as mean ± SEM (n=3). *$P < 0.05$, **$P < 0.01$.

**Supplementary Table**

Table 1 Primer sequences and product size designed for RT-qPCR

| Target gene | Forward or Reverse | Primer sequence（5 ′-3 ′） |
|---|---|---|
| *β-actin* | Forward primer | TCTGGCACCACACCTTCTA |
| | Reverse primer | AGGCATACAGGGACAGCAC |
| *HK2* | Forward primer | GATGAAGGTGGAAATGGA |
| | Reverse primer | CCTGTGGGATGGAGTAGA |
| *PFK1* | Forward primer | TGCCGCTGTTCGCTCTAC |
| | Reverse primer | AGCCTCCTCAATCTGACCTTT |
| *GAPDH* | Forward primer | GCCGAACACAAGAAGCTGGAAG |
| | Reverse primer | GGCAAATCCTGCTACGAGCACT |
| *PDH* | Forward primer | GGTGCAGTTGACAGTTCGTG |
| | Reverse primer | CTGAGATGGGGGTGTCGATG |
| *SDH* | Forward primer | TCGACAGGGGAATGGTTTGG |
| | Reverse primer | GGACTCCTTCCGAGCTTCTG |
| *G6PDH* | Forward primer | GCAAGCGTAATGAGCTGGTC |
| | Reverse primer | GGTCCTCCCTGTTGAACCTT |
| *PPAR- γ* | Forward primer | CATCAGGTTTGGGCGGGAT |
| | Reverse primer | TTTCCTGTCAAGATCGCCCT |
| *LIPA* | Forward primer | GACCACTCCCGATGCAACTC |
| | Reverse primer | GACCGAGTGTTCCTCACCAG |
